# Unraveling the urban climate crisis: Exploring the nexus of urbanization, climate change, and their impacts on the environment and human well-being – A global perspective

**DOI:** 10.3934/publichealth.2024050

**Published:** 2024-08-27

**Authors:** Sumanta Das, Malini Roy Choudhury, Bhagyasree Chatterjee, Pinanki Das, Sandeep Bagri, Debashis Paul, Mahadev Bera, Suman Dutta

**Affiliations:** 1 School of Environment and Disaster Management, Ramakrishna Mission Vivekananda Educational and Research Institute, Kolkata 700103, West Bengal, India; 2 The University of Queensland, St Lucia, Queensland 4072, Australia; 3 ICAR-Central Institute for Cotton Research, Regional Station, Sirsa, India; 4 Department of Genetics and Plant Breeding, Ramakrishna Mission Vivekananda Educational and Research Institute, Kolkata 700103, West Bengal, India

**Keywords:** urbanization, climate change, environment, human well-being, urban climate crisis, nexus

## Abstract

The accelerating pace of urbanization, coupled with the intensifying impacts of climate change, poses unprecedented challenges to both the environment and human well-being. In this review, we delved into the intricate interaction between climate change and urbanization and the various effects they have on the environment and human well-being, shedding light on the emergent urban climate crisis. Urban areas serve as epicenters for diverse socio-economic activities, yet they also contribute significantly to global greenhouse gas emissions and environmental degradation. Through an interdisciplinary lens, we explored the root causes of the urban climate crisis, examining how rapid urbanization exacerbates climate change and vice versa. By synthesizing current research and case studies, we elucidate the various environmental and social ramifications of this nexus, ranging from urban heat island effects to heightened vulnerability to extreme weather events. Furthermore, we delve into the unequal distribution of climate risks within urban populations, highlighting the disproportionate burden borne by marginalized communities. Finally, the chapter presents strategies and interventions for mitigating and adapting to the urban climate crisis, emphasizing the imperative of holistic and equitable approaches that prioritize both environmental sustainability and human well-being. Overall, this review calls for concerted efforts to unravel the complexities of the urban climate crisis and forge a path toward resilient, sustainable, and equitable urban futures.

## 1. Introduction

Understanding the urban climate crisis is paramount in addressing the intricate problems brought up by increasing urbanization and climate change [1]. Cities are at the forefront of both contributing to and experiencing the impacts of climate change, with urban areas accounting for a significant portion of global greenhouse gas emissions and facing heightened risks from extreme weather events, heat waves, and rising sea levels. Recent research underscores the urgency of this issue, with studies indicating that urban areas are projected to face increasingly severe heat waves due to the urban heat island effect, exacerbating heat-related health risks for vulnerable populations [2],[3]. Furthermore, the concentration of infrastructure and populations in urban centers heightens the susceptibility of cities to climate-related disasters such as flooding and storm surges [4]. Mitigating the urban climate crisis requires interdisciplinary approaches that integrate urban planning, sustainable design, green infrastructure, and community engagement [5]. By understanding the complexities of the urban climate crisis and implementing proactive strategies, cities can become more resilient, sustainable, and equitable in the face of climate change challenges.

Addressing urbanization and climate change is complex due to its multifaceted implications on global sustainability and human well-being. Urbanization, marked by the rapid growth of cities, increases energy consumption, waste generation, transportation demands, and infrastructure development, all of which contribute to heightened greenhouse gas emissions, resource depletion, and environmental degradation [6]. According to the Intergovernmental Panel on Climate Change (IPCC), cities are responsible for approximately 70% of global carbon dioxide emissions, highlighting the critical role urban areas play in exacerbating climate change [7]. Moreover, climate change exacerbates existing urban challenges, such as heat stress, water scarcity, and extreme weather events, disproportionately impacting vulnerable populations residing in urban areas [8]. Failing to mitigate the adverse effects of urbanization on climate change could lead to dire consequences, including increased social inequality, economic instability, and ecological collapse [9]. Therefore, concerted efforts to address urbanization and climate change are imperative to foster sustainable development, enhance resilience, and secure a habitable future for future generations.

The nexus of urbanization and climate change presents a complex web of challenges with profound implications for both the environment and human well-being. Urbanization and/or unplanned expansion of cities have been identified as a significant driver of climate change, land use changes, and the urban heat island effect [10],[11]. Conversely, climate change exacerbates the vulnerabilities of urban areas through extreme weather events, sea-level rise, and altered precipitation patterns [12]. These intertwined processes pose considerable risks to ecosystems, biodiversity, and public health. For instance, urban expansion often encroaches upon natural habitats, leading to habitat loss and fragmentation, threatening biodiversity and ecosystem services essential for human well-being [13]. Additionally, the urban heat island effect exacerbates heat-related illnesses and mortality, particularly among vulnerable populations in densely populated cities [14]. Furthermore, climate change-induced events such as flooding and storms can disrupt urban infrastructure, compromise water quality, and increase the risk of vector-borne diseases, thereby compromising human health and resilience [15]. Addressing the interconnected challenges of urbanization and climate change necessitates integrated approaches that prioritize sustainable urban planning, green infrastructure development, and climate adaptation strategies to safeguard both the environment and human well-being.

In this review, we provide insights into a critical and multifaceted issue at the intersection of urban development, climate change, and human welfare. We aim to comprehensively analyze the intricate relationships between urbanization, climate change, and their consequential impacts on both the environment and human well-being from a global perspective. Our objectives are to synthesize existing research on the dynamic interplay between urban growth and climate change, to assess the environmental and health implications of this nexus, and to identify effective mitigation and adaptation strategies. This novelty lies in its holistic approach to integrating diverse, multidisciplinary perspectives, offering a comprehensive understanding of the urban climate crisis. By bridging gaps in current research, the review highlights critical areas for policy intervention and future study, ultimately contributing to more sustainable urban development and safeguarding human well-being in a changing climate.

## 2. Interconnection of urbanization and climate change

**Figure 1.**
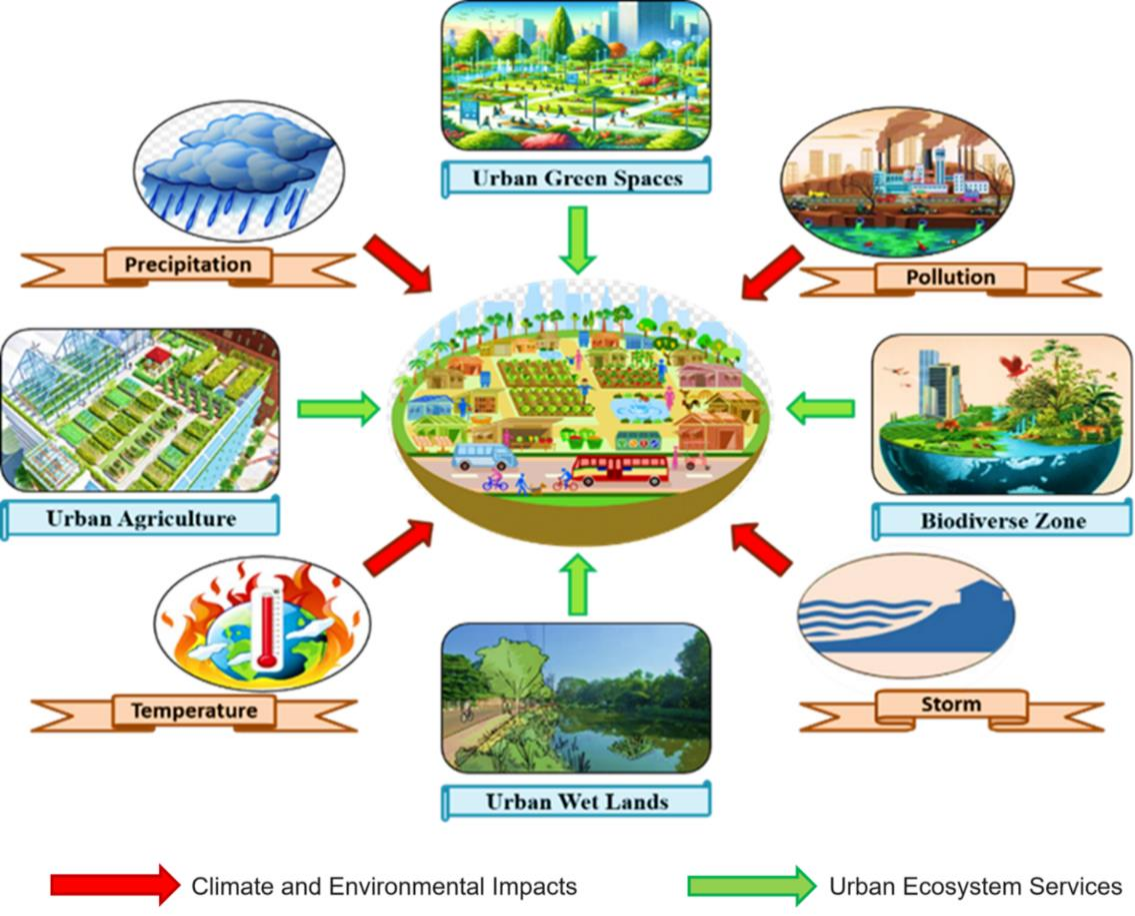
Interconnections between urban ecosystem services (depicted by green arrows) and climate change and environmental effects (depicted by red arrows).

Around seven out of ten people on Earth live in urban settings, up from 56% (4.4 billion) in 2050 [16],[17]. With cities accounting for over 80% of the global GDP and, when managed well, encouraging productivity and innovation, urbanization may lead to sustainable growth [18]. The complex interplay between urban ecosystems and climate change has gained prominence in the global environmental debate in this period of rapidly urbanizing populations and intensifying climate change. The built environment, green areas, and a variety of human activities in cities provide a range of ecosystem services that improve the resilience and well-being of city people. In addition to providing provisions like food and water, urban ecosystems provide regulatory functions, including temperature control, air quality maintenance, and stormwater management [19]. There is a complicated and dynamic link between urban ecosystem services and climate change (Figure 1).

### 2.1. Urbanization trends and patterns worldwide

**Figure 2.**
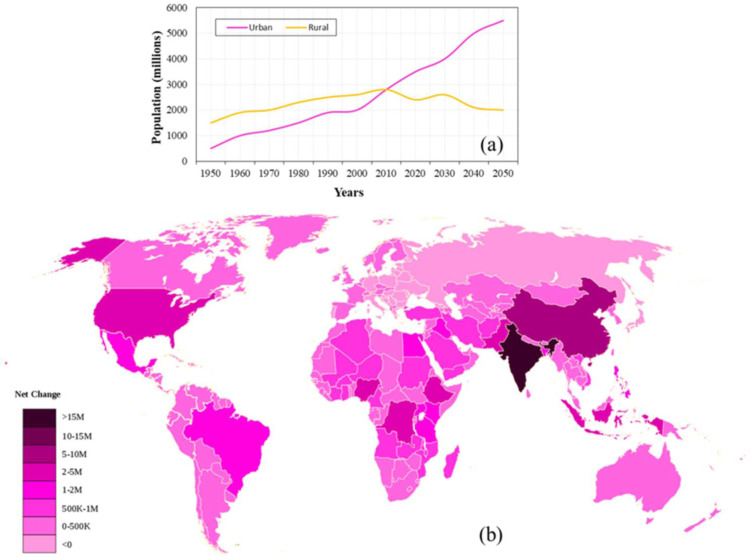
(a) Global population trends and projections from 1950 to 2050; (b) Spatial distribution of the net change in the global population from 1950 to 2016 (data source: United Nations Department of Economic and Social Affairs, Population Division 2018) [21].

Urbanization trends and patterns worldwide reflect dynamic shifts in population distribution, economic development, and social dynamics. These trends are influenced by many factors, including industrialization, rural-to-urban migration, globalization, and government policies. Examining these trends provides insight into the diverse trajectories of urban growth and development across different regions. Many developing countries are experiencing rapid urbanization as people from rural areas move to cities for greater employment prospects. This phenomenon is particularly pronounced in regions like Asia and Africa. Figure 2a depicts the growth trend and projections of rural and urban populations in the selected areas of Asia and Africa from 1950 to 2050, where a significant shift in population from rural to urban areas was observed after 2005, leading to urban expansion. For example, China has witnessed unprecedented urban growth in the last few decades, with massive urban agglomerations like the Pearl River Delta and the Yangtze River Delta emerging as global economic powerhouses [20]. Likewise, in Africa, countries like Nigeria and Kenya are experiencing significant urban growth driven by rural-to-urban migration and natural population increase [21]. India is experiencing rapid urbanization, with its urban population projected to reach 600 million by 2031 [22]. Natural population growth, migration from rural to urban areas, and economic possibilities in cities are some causes of this rise. On the other hand, developed regions like North America and Europe have experienced extensive urban growth and sprawl, leading to challenges such as traffic congestion, pollution, and loss of green spaces [23],[24]. For example, suburbanization in the United States and cities like Los Angeles and Atlanta have witnessed significant suburban expansion, resulting in sprawling metropolitan areas [24],[25]. In contrast, urbanization in India is often characterized by unplanned growth, leading to urban sprawl and the proliferation of informal settlements or slums. These settlements lack basic amenities and are often located in hazardous areas such as floodplains or steep slopes. Dharavi in Mumbai, which is one of the largest slums in Asia, the Delhi-NCR region, has witnessed significant urban growth and sprawl [26],[27]. Figure 2b shows the net change in the global population distribution from 1950 to 2016, where India stands highest among the countries.

### 2.2. Factors affecting urbanization and climate change

Urbanization and climate change are driven by a multitude of interconnected factors, ranging from demographic shifts and economic development to energy consumption and land-use changes, such as Population growth, Industrialisation and economic development, Energy consumption and fossil fuel use, Land use changes and deforestation, Urban planning and infrastructure, etc. Urbanization and climatic change are significantly fueled by population increase. Urban areas are growing due to increased demand for resources, infrastructure, and housing as the world's population rises. This leads to increased emissions of greenhouse gases, as well. By 2050, it is expected that there will be 9.7 billion people on the planet, most of them living in cities [21]. Additionally, industrialization and economic development drive urbanization by creating job opportunities and attracting people to urban centers. However, industrial activities also contribute significantly to the emissions of greenhouse gases from the burning of fossil fuels and industrial processes. For example, China's rapid industrialization has led to massive urban growth, particularly in cities like Shanghai and Beijing, while also contributing to high levels of carbon emissions [28]. Industrial activities, including manufacturing and construction, are significant sources of emissions of greenhouse gases in India. As industries grow, so does their carbon footprint, contributing to climate change [29]. Furthermore, generating energy exclusively from fossil fuels and transportation is a major driver of climate change. Urbanization increases energy demand, leading to higher emissions of greenhouse gases unless renewable energy sources are prioritized. For example, the United States, with its high energy consumption per capita, contributes significantly to global carbon emissions due to its reliance on fossil fuels for electricity and transportation [30]. The burning of coal, oil, and natural gas releases greenhouse gases, contributing to global warming, and India is one of the world's largest consumers of coal for electricity generation, leading to high carbon emissions [31]. Urbanization often involves converting natural landscapes into built environments, leading to deforestation and habitat loss. Deforestation contributes to climate change by reducing carbon sinks and releasing stored carbon into the atmosphere. For example, the Amazon rainforest, one of the world's largest carbon sinks, is rapidly cleared for agriculture, urban expansion, and infrastructure development, contributing to increased carbon emissions [32]. Deforestation for agriculture, infrastructure, and urbanization is a significant issue in India. Urbanization and agricultural conversion of forests increase carbon emissions and biodiversity loss [33]. Besides, urban planning and infrastructure decisions influence the emissions intensity and resilience of cities. Poorly planned urbanization can lead to inefficient transportation systems, energy-intensive buildings, and vulnerability to climate-related hazards. Sustainable urban planning techniques have been adopted by cities like Copenhagen, Denmark, by including substantial public transit networks, bike-friendly infrastructure, and green spaces, leading to lower emissions per capita and enhanced ability to adapt to climate change [34]. Alternatively, infrastructure development, including transportation networks, housing, and utilities, influences urbanization in developing nations such as India. Improved infrastructure attracts people to urban areas and facilitates economic growth. The development of metro rail systems in cities like Delhi and Kolkata has improved connectivity and accessibility, further fueling urbanization [35].

### 2.3. Feedback Loops: How does urbanization influence climate change and vice versa?

Urbanization and climate change are intricately linked, each influencing and exacerbating the other in multifaceted ways. As the global population continues to migrate towards urban areas, cities are expanding at unprecedented rates, leading to increased energy consumption, heightened demand for resources, and intensified greenhouse gas emissions [10]. This rapid urbanization significantly contributes to climate change through activities such as industrial production, transportation, and reliance on fossil fuels for energy generation [7]. For instance, megacities in Europe and Asia, including Tokyo, Osaka, Seoul, Beijing, and Mumbai; North and South American megacities, including New York, Los Angeles, Mexico City, Sao Paulo, and Buenos Aires, are significant contributors to carbon dioxide emissions due to industrial activities, transportation, and energy consumption [36]. Moreover, urbanization alters land surfaces, replacing natural ecosystems with built environments, which reduces carbon sinks and exacerbates the urban heat island effect, leading to higher local temperatures. Conversely, climate change poses numerous challenges for urban environments, amplifying existing vulnerabilities and introducing new risks. The urban heat island effect is exacerbated by rising temperatures, leading to more frequent and intense heat waves that endanger public health and strain infrastructure [3]. Furthermore, shifting precipitation patterns and rising sea levels increase the likelihood of flooding in coastal cities, threatening lives, property, and critical infrastructure [37]. Droughts and water scarcity, exacerbated by climate change, strain water resources in urban areas, jeopardizing access to clean water for millions [38]. The condition is dire, with urban areas facing the dual challenge of being significant contributors to climate change while simultaneously bearing the brunt of its impacts [6]. Without decisive action, the consequences of this interconnection will only worsen, jeopardizing the sustainability and resilience of cities worldwide. Urgent efforts are needed to mitigate emissions, enhance adaptive capacity, and integrate climate resilience into urban planning and development strategies [12]. Collaboration among governments, communities, and stakeholders is essential to address the complex and interconnected challenges posed by urbanization and climate change and to build sustainable, resilient cities capable of withstanding future uncertainties.

## 3. Impacts of urbanization on climate

Urban centers accelerate the transformation of land, consume large amounts of energy, and release greenhouse gases (GHGs), all of which contribute to global warming [39],[40]. In addition, various factors, including city planning, urban layout, economic activity and pattern, population growth and size, customs and culture, variations in weather patterns, and more, influence how cities affect the surrounding environment [41]–[43]. Regulating services, which are essential to urban resilience, include invisible but vital tasks including pollinating plants, controlling floods and diseases, and preserving the quality of the air and soil [44],[45]. Urban vegetation, especially trees, absorb carbon dioxide through biomass storage and photosynthesis, so directly reducing greenhouse gas emissions [46]–[48]. However, the development and survival of plants and trees may be impacted by changing climatic circumstances. Increasing temperatures may dry and injure roots, decreasing their capacity to absorb water, influencing soil microbial populations, and influencing plant intake of nutrients [49]. Urban trees not getting enough nutrients may develop more slowly, become unstable, and be more susceptible to other influences. Thus, the ability of urban green areas to mitigate local heat was reduced by the climate change-related increase in temperature. The effects of heat stress are one component of climate change that sticks out among the others. Stress not only impacts foraging behavior, pollinator growth overall, and pollination services, but it also causes noticeable alterations in these domains [50]. As climate change advances, these detrimental consequences are anticipated to become even more evident, with notable changes anticipated in tropical and temperate locations [17]. Heat waves become more frequent and longer due to climate change and act as the primary driver causing these changes [51]. The effects on tropical areas, which are already acclimated to high temperatures, are especially concerning. It is anticipated that these tropical regions would be negatively impacted more than temperate locations due to the predicted temperature rises brought about by climate change [52].

### 3.1. The urban heat island effect

Anthropogenic carbon dioxide emissions were mostly produced in metropolitan areas by a combination of factors such as the fast growth of impermeable surfaces, altering land uses, industrial processes, transportation, and high energy consumption [53]. The phenomenon known as the urban heat island (UHI) effect is caused by a combination of factors that raise air temperatures in metropolitan regions relative to their rural surroundings [54]. The altered temperature gradients in urban areas, with higher daytime temperatures and reduced cooling at night compared to rural areas, are the characteristics of the UHI effect.

The UHI effect is predominantly a result of human activities, with urbanization leading to the replacement of natural landscapes (vegetation and green spaces) with impervious surfaces (concrete, asphalt, and buildings), which absorb and emit heat, contributing to elevated temperatures in cities [54]. For example, in cities like Los Angeles, USA, extensive paving and construction have transformed natural landscapes into heat-absorbing surfaces, leading to significant UHI intensification [55]. On the other hand, rapid urbanization in Indian cities like Mumbai and Delhi has led to the widespread conversion of green spaces and agricultural land into concrete jungles, exacerbating UHI effects [56]. The scarcity of vegetation in urban areas further exacerbates the UHI effect, as green spaces provide shading and evaporative cooling [57]. For example, cities in arid regions like Phoenix, USA, and rapidly developing Indian cities like Chennai, Ahmedabad, etc. face similar challenges with declining green cover and inadequate urban forestry, exacerbating UHI effects [58]. Furthermore, The layout and design of urban areas can impact the UHI effect. Tall buildings, narrow streets, and lack of open spaces can restrict airflow and create “urban canyons” that trap heat and reduce ventilation. This can lead to the accumulation of heat and pollutants, further exacerbating the UHI effect. For example, European cities like London, UK, with dense urban cores, experience pronounced UHI due to limited green spaces and high building densities [57]. Likewise, Indian cities such as Kolkata and Bangalore exhibit UHI effects influenced by high population density and compact urban layouts, exacerbating heat accumulation [58]. Moreover, human activities such as industrial processes, transportation, energy consumption, and waste heat from buildings contribute to heat generation in urban areas. For example, Tokyo in Japan and Indian megacities, New Delhi and Mumbai, experience significant UHI effects attributed to the concentration of industrial activities and high population density [59]. UHI effect can have a range of consequences on the environment, public health, infrastructure, and overall quality of life in urban areas. Some of the key consequences of the UHI effect include:

#### 3.1.1. Increased energy consumption

The UHI effect can drive up energy consumption for air conditioning and cooling systems in buildings, leading to higher electricity bills and increased greenhouse gas emissions. This creates a feedback loop where higher energy demand contributes to further warming in urban areas. According to a study, urban areas account for approximately 75% of global energy consumption and greenhouse gas emissions, with a significant portion attributed to cooling demands in response to urban heat islands [60]. For example, In India, cities like Delhi and Mumbai witness a surge in electricity demand during summer months due to the increased use of air conditioning to combat the urban heat island effect [61].

#### 3.1.2. Health impacts

The UHI effect may be a factor in the increased prevalence of heat-related disorders such as dehydration, heat exhaustion, and heatstroke. During extreme heat events, vulnerable populations, such as the elderly, children, and people with pre-existing health conditions, are more vulnerable [62]. Situating in an extremely hot and humid tropical climatic region, Indian megacities like Kolkata and Chennai experience severe heat waves during summer months, contributing to heat-related illnesses and mortality rates among vulnerable populations [63].

#### 3.1.3. Environmental degradation

Elevated temperatures in cities can worsen air quality by promoting the formation of ground-level ozone and other pollutants. Higher temperatures in urban areas can increase the frequency and intensity of rainfall events, leading to flash floods and stormwater runoff. Impervious surfaces in cities prevent water from infiltrating the ground, resulting in urban flooding and water pollution [64].

#### 3.1.4. Water stress

Urbanization can strain water resources through increased demand for drinking water, wastewater generation, and stormwater runoff. Research indicates that urban areas with intense heat island effects experience higher water demand for irrigation, landscaping, and domestic use, leading to increased water stress in regions already facing water scarcity [65]. For example, Indian cities like Jaipur and Ahmedabad face water stress exacerbated by the urban heat island effect, with higher water demand for residential and industrial cooling contributing to strained water resources [66].

#### 3.1.5. Social inequities and economic cost

The unequal distribution of climate risks within urban populations is a critical issue that is exacerbated by socio-economic disparities. Vulnerable communities, including low-income neighborhoods and marginalized groups, often reside in areas with higher exposure to climate hazards such as flooding and/or extreme heat and are disproportionately affected due to factors such as inadequate access to cooling resources, limited green spaces, and higher exposure to heat-related health risks. Social inequities can be exacerbated by the unequal distribution of urban heat impacts. Urban heat islands impose economic burdens on cities through increased healthcare costs, reduced labor productivity, and damage to infrastructure [67]. According to a recent report, the economic costs associated with heat-related illnesses and productivity losses in urban areas are projected to rise significantly in the coming decades, particularly in developing countries [68]. For example, the economic costs of the urban heat island effect in Indian cities include increased healthcare expenditures, loss of agricultural productivity, and damage to infrastructure from extreme heat events [68].

### 3.2. Alteration of local climate patterns and implications for extreme weather events in urban areas

Urbanization influences local temperature patterns, which alters temperature gradients and can lead to changes in regional climate dynamics [54],[57]. For example, a study conducted in cities like Beijing, China, has shown significant temperature increases attributed to UHI effects, impacting regional climate patterns [69]. Moreover, urbanization can alter local precipitation patterns by disrupting natural water cycles. Impervious surfaces in cities prevent water from infiltrating the ground, leading to increased surface runoff and the risk of urban flooding [65]. For example, a study conducted in Atlanta, USA, has shown altered precipitation patterns due to urbanization, with increased intensity and localized effects [70].

**Figure 3.**
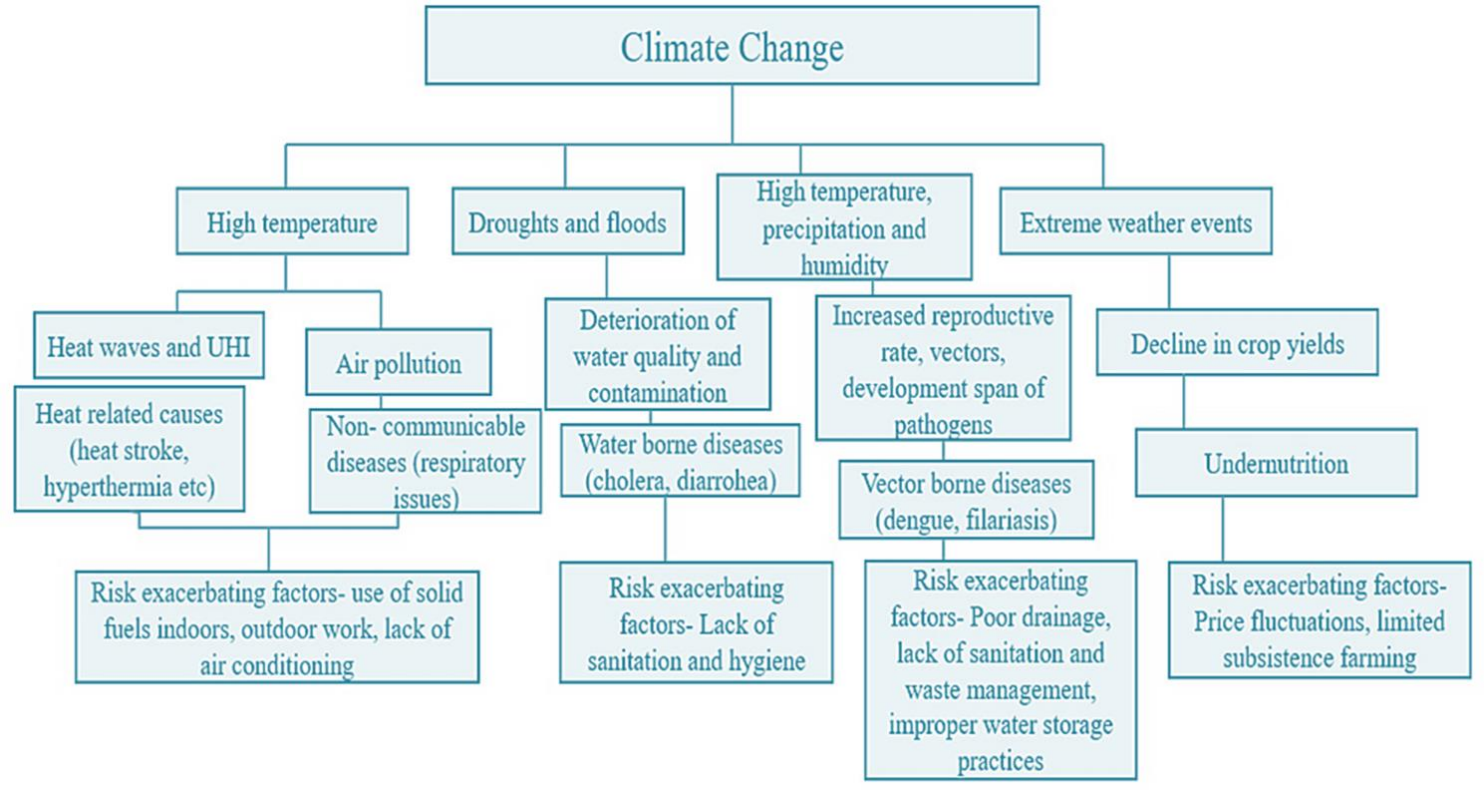
Flow chart showing the alteration of local climate patterns and associations between climate change and the health of the urban areas

Likewise, urbanization in highly populated Indian megacities like Kolkata and Chennai has been linked to altered monsoonal patterns and localized changes in precipitation distribution [56]. In addition, research indicates that urban centers are major contributors to global CO_2_ emissions, with cities like New York, USA, emitting substantial amounts of greenhouse gases [71]. Furthermore, urbanization leads to changes in land cover, surface properties, and ecosystem services [57]. For example, cities like Sao Paulo, Brazil, have witnessed significant changes in land use patterns associated with urban expansion, impacting local climate patterns and ecological systems [72]. Indian cities also experience rapid land use changes, converting agricultural land and green spaces into urban areas, leading to altered microclimates and environmental degradation [56],[59]. On the other hand, alterations in local climate patterns can lead to a rise in the frequency and severity of extreme weather phenomena, including heat waves, droughts, storms, hurricanes, and heavy rainfall episodes. These events can significantly impact communities, infrastructure, agriculture, and natural ecosystems. For instance, sea level rise and storm surges, exacerbated by urbanization and coastal development, pose a threat to coastal megacities situated along the Atlantic coast of the United States, southeast Asia, and islands in the Indo-Pacific and Caribbean [73]. Likewise, Indian coastal megacities such as Mumbai, Chennai, Kolkata, etc., are prone to extreme weather events, including cyclones, urban floods, and heavy rainfall, which can cause widespread damage and disruptions, particularly in densely populated urban areas [74],[75]. Figure 3 depicts the interlinkage of altering local climate patterns and its implications for extreme weather events in urban areas.

## 4. Climate change impacts on urban environments

Climate change impacts on the urban environment are multifaceted and profound, presenting challenges that extend beyond traditional environmental concerns. Increased frequency and severity of extreme weather events on a local to global scale, especially in poor nations and coastal regions, have been observed as repercussions of climate change [40]. Table 1 summarizes a few pieces of literature showing the impacts of climate change on urban environments across different countries.

**Figure 4.**
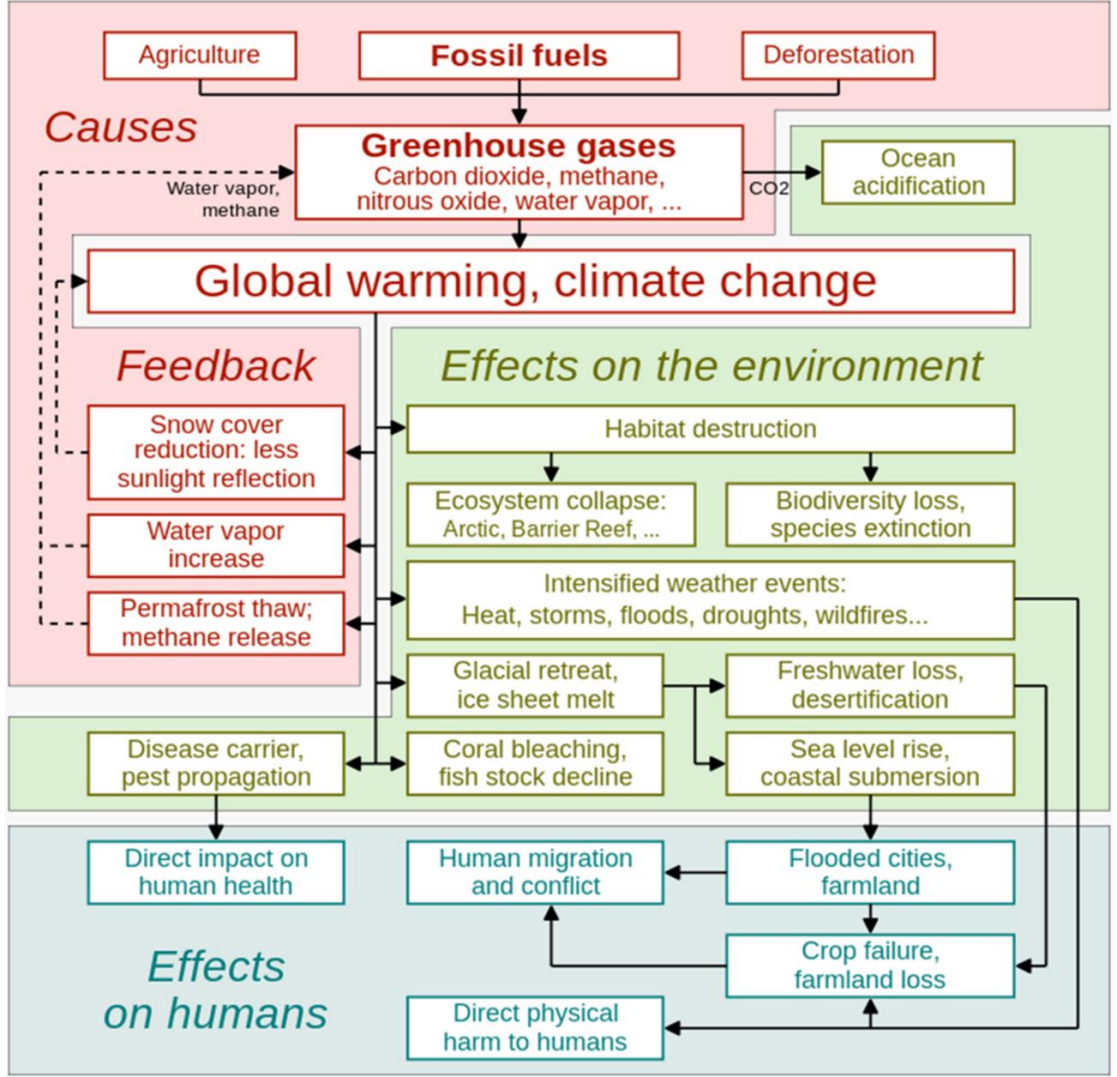
A block diagram depicting the major contributors to global warming and climate change as well as its causes and extensive impacts on the urban environment and humans, including feedback.

Variations in rainfall patterns can threaten human health and energy needs, while heat waves and the effects of urban heat islands can strain stormwater management systems and result in flooding. Furthermore, the resilience and sustainability of urban ecosystems can be negatively impacted by climate change, which can also alter ecological processes and biodiversity [17]. For example, wetland ecosystems in coastal towns are threatened by increasing sea levels [78]. Furthermore, urban vegetation yields are impacted by changes in temperature and precipitation patterns, which can result in heat stress and decreased primary productivity [80],[84]. Elevated temperatures may also affect the chemical composition of wood, influencing its resilience against rot, vermin, and mold [83]. Drought or excessive rainfall brought on by changes in precipitation impacts water resources and food production in urban and peri-urban areas [81]. Changes in precipitation patterns reduce the amount of water available, which affects sanitation, hydration, and the spread of waterborne illnesses. The growth of feed crops is impacted by climate change, which also affects animal health and nutrition. It also changes the distribution of diseases and pests, necessitating the use of more resources [86]. Furthermore, data indicates that the ongoing effects of climate change are causing a shift in the geographical distribution of insect pollinators. It is anticipated that this phenomenon will continue, mostly impacting butterflies and bumblebees [17]. Figure 4 summarizes the primary contributors to global warming and climate change as well as its causes and extensive impacts on urban environments. Overall, the global urban environments are adversely affected by climate change through rising temperatures and heat stress, changing precipitation patterns more frequent flood risks, and sea level rise and increasing vulnerability of the coastal cities. Addressing these impacts requires integrated strategies encompassing sustainable urban planning, green infrastructure development, community resilience building, and equitable access to resources, ensuring urban areas can withstand and adapt to the challenges of a changing climate.

**Table 1.** Climate change impacts on urban environments.

**S.N.**	**Climatic variable**	**City/Area**	**Effect on the urban environment**	**References**
1.	Rise in Temperature	Africa and Asia continent	Change in precipitation pattern	Anand & Seetharam [40]
2.	Variations in rainfall patterns	USA	Flooding	Weiskopf et al. [76]
3.	Increasing sea levels	Coastal towns of San Francisco	Threats to wetland ecosystems; carbon sequestration	Plane et al. [77]; Speak et al. [78]
4.	Rise in temperature, Heat waves	Bucharest, Romania, and Leipzig, Germany	Heat stress; Urban heat island formation	Carlan et al. [79]
5.	Changes in precipitation patterns	Leipzig, Germany	Decreased urban productivity	Kabisch et al. [80]
6.	Changes in precipitation	Leipzig, Germany	Droughts, floods in urban areas	Haase & Hellwig [81]
7.	Changes in precipitation patterns	East Africa	Effect on sanitation, hydration, and the spread of water-borne illnesses	Bett et al. [82]
8.	Elevated temperatures and extended heat waves	Mississippi	Stress on urban tree foliages	Ayanleye et al. [83]
9.	Variations in precipitation patterns	Florida	Hurricanes and storms;	Landry et al. [84]
10.	Changes in precipitation pattern	Kampala, Uganda	Droughts, floods	Sabiiti et al. [85]

### 4.1. Rising temperatures and heat stress in cities: Impacts

**Figure 5.**
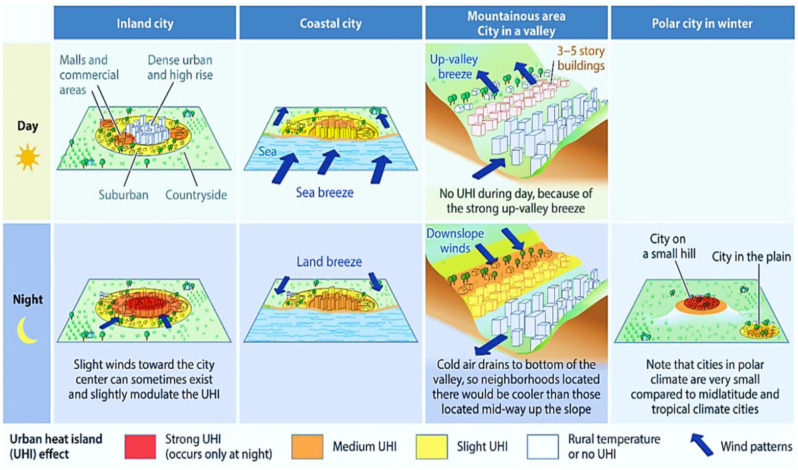
A pictorial overview of the UHI formation in various geographic contexts, including coastal cities, hilly cities, inland cities, and polar cities during the winter and its day and night time variations (*Adapted from* Masson et al. [92]).

Rising temperatures in cities, aggravated by climate change and urban heat island effect, pose significant challenges in terms of heat stress. This leads to higher temperatures in cities, especially during heatwaves, which can harm people's health, especially for vulnerable groups, including the elderly, kids, and low-income communities [87]. Usually, the albedo differences between urban areas and rural countryside are typically minimal in most cities. Schwarz & Manceur [88] reported a range of urban-rural albedo differences from -0.09 to +0.03, with an average suggested value of -0.05. While urban temperatures are more responsive to changes in albedo during the daytime [89], the actual contrast in albedo between urban and rural areas is not substantial. Despite individual surfaces within urban environments often possessing albedo values of 0.30 or higher, such as light-colored walls, the reflected energy gets absorbed by other urban surfaces through radiative trapping [90]. Consequently, this limits the proportion of energy reflected in the sky and thus constrains the overall urban albedo [91]. Figure 5 conceptualizes the UHI formation in various geographic contexts, including coastal cities, hilly cities, inland cities, and polar cities during the winter and its day and night time variations. Inland metropolitan suburbs, particularly industrial and commercial zones with vast parking lots, are frequently warmer during the day than their rural counterparts. Because buildings obstruct the sea air, coastal communities can have daytime temperatures warmer than those of the surrounding interior cities, although they are normally colder. Urban centers tend to be warmer than rural areas, with high UHI throughout the night when the weather is quiet. Orographic flows can make cities in valleys an exception. Hills can also influence how strong the nocturnal UHI is, particularly on chilly, clear nights [92].

Rising temperatures and heat stress in cities have significant and multifaceted impacts across various aspects of urban life, such as heat stress contributing to a higher incidence of heat-related illnesses such as heat exhaustion, heatstroke, and dehydration [93]. Additionally, rising temperatures increase energy demand for cooling, leading to higher electricity consumption and greenhouse gas emissions that can increase air pollution and worsen air quality, especially in cities where heat-related energy use relies heavily on fossil fuels [94]. Furthermore, heat stress disproportionately affects socioeconomically disadvantaged communities and neighborhoods with limited access to air conditioning, green spaces, and cooling centers [67]. This exacerbates existing social inequities, as vulnerable populations may face greater challenges in coping with extreme heat events. High temperatures can reduce the lifespan of infrastructure elements like buildings, bridges, and transportation systems by buckling roads and railways, expanding materials like concrete and metal, and straining electrical grids, which may adversely affect the urban economy [95]. Additionally, rising temperatures and heat stress impact urban ecosystems, leading to changes in plant and animal species distribution and reduced biodiversity [17].

### 4.2. Intensification of precipitation and flood risks: Impacts

In recent years, there has been an increase in natural disasters, notably floods associated with extreme precipitation events in cities [96]. Precipitation extremes are expected to increase in frequency, spread, and intensity during the 21st century, disproportionately affecting cities [97]. Although previous research has emphasized how heavy precipitation is affected by global warming, little is known about how extreme precipitation will change in the future at small spatiotemporal scales, particularly in cities. Overall, the intensification of precipitation and associated flood risks have profound impacts on various aspects of urban environments, such as intense precipitation events can cause flooding that damages infrastructure such as roads, bridges, buildings, and utilities, leading to economic loss. Additionally, urban floods pose significant health and safety risks to individuals and communities. They can lead to waterborne diseases, contamination of drinking water sources, exposure to hazardous materials, and physical injuries or fatalities [98]. Floods associated with intensified precipitation can cause environmental degradation, including soil erosion, sediment runoff into waterways, and pollution of natural habitats. Floodwaters may carry contaminants such as chemicals, sewage, and debris, affecting water quality and ecosystem health [99]. Furthermore, vulnerable communities, including low-income neighborhoods, marginalized populations, and areas with inadequate flood protection infrastructure, are disproportionately affected by intensified precipitation and flood risks.

### 4.3. Sea level rise and increasing vulnerability to the coastal cities

**Figure 6.**
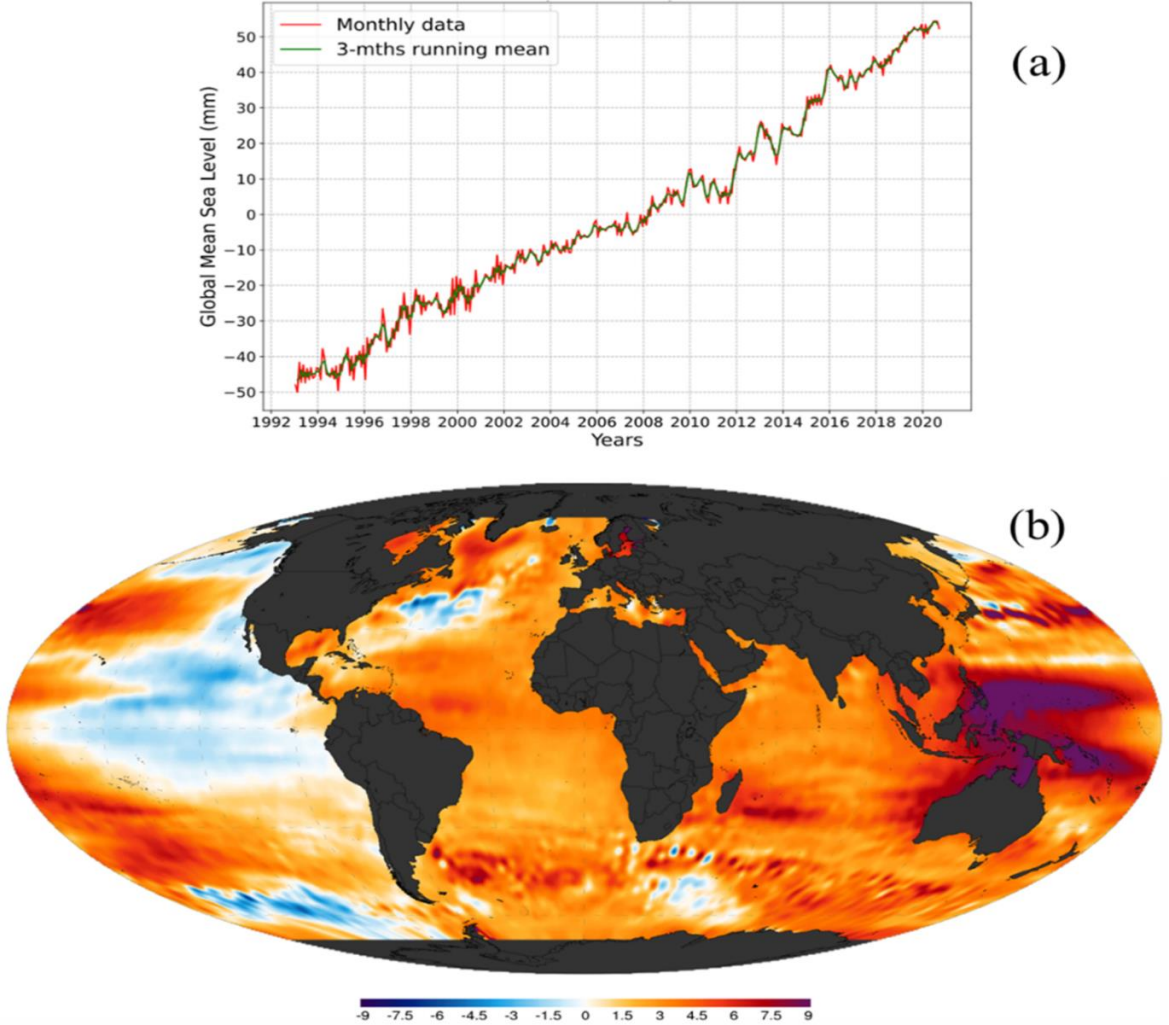
(a) Graphical trend in global mean sea level (mm) from 1993–2020 showing a steady positive increment in global sea level (Data source: White [102], Commonwealth Scientific and Industrial Research Organisation (CSIRO), Accessed on February 25, 2024); (b) Sea-level trend spatiotemporal map across the word from 1993 to 2020. The color scale bar indicates negative (blue shades) to positive (brown shades) trend in mm/year of global sea level (data source: National Oceanic and Atmospheric Administration (NOAA) global sea level data, Accessed on February 25, 2024) [105].

Sea level rise, driven by climate change, presents significant vulnerabilities and adaptation challenges for coastal cities. As global temperatures increase, glaciers and polar ice caps melt, causing the ocean to expand and subsequent rise in sea levels. Coastal cities, with their dense populations and critical infrastructure near coastlines, are particularly vulnerable to the impacts of sea level rise [100]. These impacts include increased hazards to coastal ecosystems and biodiversity, greater floods during storms, and saltwater intrusion into freshwater sources. Coastal erosion is one of these effects [101]. Data have suggested, since 1993, that the global mean sea level has increased by 100 mm (-50 mm to +50 mm), and the average annual increase in sea level has been estimated to be approximately +2.6 mm to +2.9 mm, with a margin of error of ±0.4 mm (Figure 6a). Figure 6b depicts the global sea-level trend (mm/yr) spatio-temporal map from 1993 to 2020. Projections suggest that by the year 2100, global average sea levels could surge by as much as 6 feet [102]. Even a rise of 1.6 feet by 2070 would imperil 150 million people worldwide and assets totaling $35 trillion [37]. These risks are particularly acute in 20 of the world's most vulnerable and rapidly expanding port cities. Earlier, Nicholls [103] proposed that the likelihood of coastal flooding will escalate due to expanding populations and the heightened economic significance of coastal cities, particularly notable in developing nations.

Sea-level rise poses a significant threat to coastal cities, leading to various challenges and vulnerabilities, such as increased coastal flooding, particularly in low-lying areas and rapidly expanding port cities. This flooding risk is exacerbated by expanding populations and the economic significance of coastal cities, especially in developing nations [100]. Additionally, sea-level rise enhances seawater intrusion into coastal aquifers, threatening freshwater resources and increasing groundwater salination [104].

## 5. Human well-being in urban climates

### 5.1. Health risks and social inequities associated with urban climate conditions - An overview

**Figure 7.**
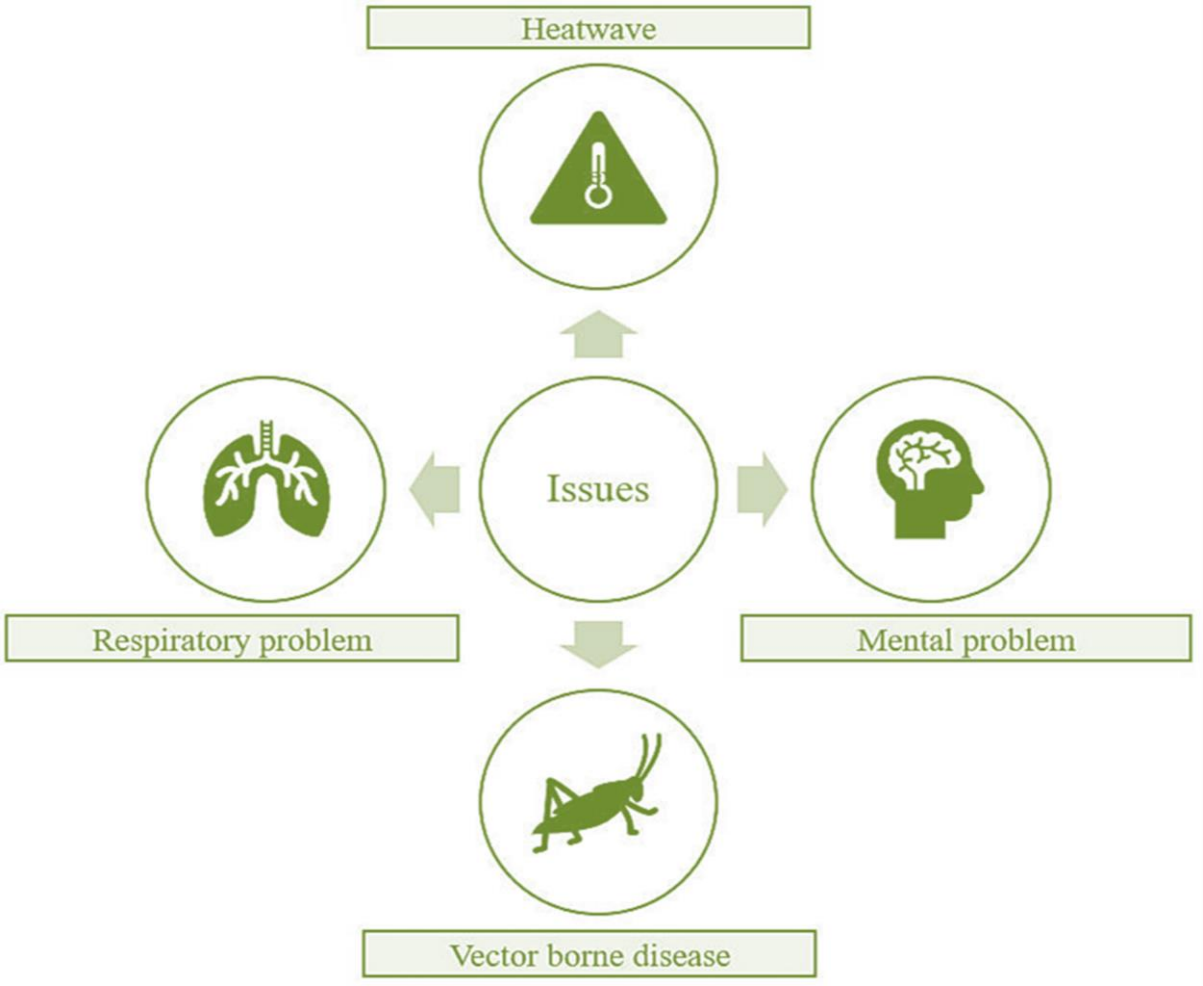
The major human health risks associated with the nexus of urbanization and climate change.

The conditions of human beings in urban climates can be both challenging and complex, influenced by a myriad of factors, including temperature variations, extreme humidity, decreased air quality, infrastructure, and socio-economic disparities [106]. These can increase heat stress among residents, particularly vulnerable populations [107]. Figure 7 depicts the major human health risks of climate change and urbanization.

Inadequate housing conditions, such as overcrowding and poor ventilation, can exacerbate the health impacts of extreme weather events, such as heat waves and flooding [106]. Additionally, air quality in urban areas is often compromised by pollutants emitted from vehicles, industrial activities, and other sources [107]. High levels of air pollution have been linked to respiratory illnesses, cardiovascular diseases, and even premature death [107]. The combination of elevated temperatures and poor air quality exacerbates the health risks faced by urban dwellers, particularly during heat waves when air pollution levels tend to peak [108]. For instance, the deadly heatwave in 2015 in India resulted in thousands of deaths, primarily among the elderly and those engaged in outdoor labor. During heatwaves in cities like Chicago, USA, and Paris, France, there is a surge in heat-related hospital admissions and mortality rates. Indian megacities like Delhi and Ahmedabad experience severe heatwaves during summer, with temperatures exceeding 45°C, resulting in numerous heat-related deaths and hospitalizations [109]. In addition, urban areas in overpopulated countries like India and China experience excessive air pollution, mostly from industrial operations, construction dust, and vehicle emissions. Lung cancer and respiratory conditions, including asthma and chronic obstructive pulmonary disease (COPD), can result from prolonged exposure to polluted air [110]. Cities with high levels of air pollution, such as Beijing, China, and Delhi, India, report increased hospital admissions for respiratory ailments during periods of poor air quality [111],[112]. Delhi frequently faces severe air quality crises, with particulate matter levels far exceeding safe limits, leading to significant public health concerns [113]. Moreover, access to green spaces and adequate housing can significantly impact the well-being of urban residents. The lack of green spaces limits opportunities for recreation and outdoor activities, exacerbating the urban heat island effect and contributing to mental health issues such as stress and anxiety [80]. For instance, researchers have reported a higher prevalence of mental health disorders among urban residents compared to rural populations, highlighting the need for comprehensive mental health interventions in urban areas [114]. Studies in cities like New York, USA, and London, UK, have found an increased frequency of mental health issues in urban as opposed to rural populations [115]. Rapid urbanization in Indian cities like Bangalore and Kolkata is also associated with increased stress and mental health disorders among residents, particularly in low-income communities [116].

Besides, urbanization creates favorable breeding grounds for mosquitoes, which raise the risk of vector-borne diseases such as dengue fever, chikungunya, and malaria. For example, in India, rapid urban growth has led to the proliferation of informal settlements with inadequate sanitation and drainage systems, creating ideal conditions for mosquito breeding. Cities in other tropical regions like Rio de Janeiro, Brazil, etc., frequently experience outbreaks of dengue fever during the monsoon season, with densely populated urban areas at higher risk. Besides, inadequate water and sanitation infrastructure in urban areas contributes to the spread of waterborne diseases such as cholera, typhoid, and gastroenteritis. Cities with poor water quality management, such as Dhaka, Bangladesh, and Chennai, India, report outbreaks of waterborne diseases during the monsoon season and following flooding events. Table 2 summarizes the findings of a few studies showing the implications of the urbanization and climate change nexus on human health and well-being across the countries.

**Table 2.** Effect of climate change on human health.

**Location**	**Responsible key Climatic variable**	**Impact on human health (Disease/Illness/ fatalities caused)**	**References**
USA, Canada, China, and Egypt	Global warming and Heat waves	Vector-borne diseases- onchocerciasis, and malaria resulted in several fatalities	Kalkstein & Smoyer [117]; Kolstad & Johansson [118]
Doha (Qatar)	Flood/ Storms	Waterborne diseases resulted in fatalities, several injuries, and property loss	Ajjur & Al-Ghamdi [119]
Fiji and Peru	Global Warming	Diarrhea	Kolstad & Johansson [118]
European & Australian cities	Temperature Rise	Salmonella	D'Souza et al. [120]
Massachusetts & Canada	Temperature Rise	Salmonella, Campylobacter & Escherichia coli	Naumova et al. [121]
Japan	Temperature Rise	Gastroenteritis	Onozuka et al. [122]
Dhaka (Bangladesh)	Temperature Rise	Noncholera Diarrhea	Hashizume et al. [123]
United States	Increased temperatures and extreme weather events	Higher rates of stress, anxiety, depression, and post-traumatic stress disorder (PTSD) among affected populations	Clayton et al. [124]
Australia	Climate change, heat stress	Rural communities are particularly vulnerable to climate change, experiencing increased heat-related illnesses, respiratory issues from bushfire smoke, and mental health impacts due to drought and resource scarcity.	Hanna & McIver [125]
India	Increased temperature and heat stress	significant increase in heat-related mortality, particularly among vulnerable populations such as the elderly and those with pre-existing health conditions. The frequency and intensity of heatwaves have been rising due to climate change.	Murari et al. [126]
Europian cities	Extreme weather events, heatwaves	Increased mortality and morbidity from heatwaves, changes in the distribution of infectious diseases, and health impacts from extreme weather events like floods	Kovats & Hajat [127]
Sub-Saharan Africa	Temperature rise, variability in precipitation	Climate change is expanding the range and seasonality of vector-borne diseases such as malaria and dengue fever, thereby increasing the disease burden	Caminade et al. [128]
Brazil	Heat stress, floods	Climate change exacerbates health risks in the Amazon basin through increased incidence of infectious diseases, malnutrition due to impacts on agriculture, and heat stress.	Confalonieri et al. [129]

Finally, socio-economic disparities also play a crucial role in determining the vulnerability of urban populations to climate-related risks. Marginalized communities, including low-income households and ethnic minorities, often bear the brunt of environmental hazards due to factors such as limited access to healthcare, housing instability, and employment insecurity [130]. For example, in cities like Sao Paulo, Brazil, informal settlements lack access to basic services like clean water and sanitation, making residents more susceptible to health risks during extreme weather events and hindering their ability to adapt [131]. Likewise, In Delhi, India, the urban poor living in informal settlements like slums are more susceptible to respiratory illnesses and heat-related illnesses during heatwaves and air pollution episodes, exacerbated by overcrowded and poorly ventilated housing conditions [111]. Furthermore, climate-related disasters and environmental degradation can trigger displacement and forced migration, particularly among marginalized communities living in hazard-prone areas such as floodplains and coastal zones. Social inequities exacerbate the vulnerability of displaced populations, who often face barriers to accessing adequate housing, livelihood opportunities, and social support networks in host communities. For example, In Dhaka, Bangladesh, residents of low-lying and flood-prone areas such as Korail Basti are at risk of displacement due to riverbank erosion and urban flooding, often resulting in informal resettlement in even more vulnerable locations. Besides, marginalized communities in cities often have limited representation and voice in decision-making processes related to urban planning, climate adaptation, and disaster risk management. This lack of participation reinforces social inequities and undermines efforts to address the needs and concerns of vulnerable populations, leading to further marginalization and exclusion [130]. For instance, in Jakarta, Indonesia, marginalized fishing communities in areas like Muara Angke have little voice in decisions regarding coastal development and climate adaptation measures, resulting in their continued vulnerability to sea-level rise and land subsidence [132]. Overall, addressing these disparities requires a multifaceted approach that integrates urban planning, public health interventions, and social equity initiatives to ensure that all residents have the resources and resilience to cope with the challenges of urban climates.

### 5.2. Economic impacts of climate extremes on urban communities

The economic impacts of climate extremes on urban communities are significant and multifaceted, affecting various sectors. Climate extremes such as floods, storms, and heatwaves can cause extensive damage to urban infrastructure, including roads, bridges, buildings, and utilities, leading to substantial economic loss [133]. For example, Hurricane Katrina, which struck New Orleans in 2005, resulted in an estimated $125 billion in damages, including infrastructure repair costs and lost economic output [134]. Additionally, climate extremes disrupt business operations in urban areas, leading to lost revenues, productivity losses, and supply chain disruptions. For instance, the 2011 floods in Bangkok, Thailand, disrupted manufacturing operations in industrial zones, leading to supply chain disruptions and economic losses estimated at $46 billion [135]. Furthermore, urban communities reliant on agriculture for food supply and livelihoods are particularly vulnerable to these impacts. For example, the 2012 drought in the United States Midwest resulted in significant crop losses, particularly for corn and soybeans, leading to higher food prices and economic losses for farmers and consumers. Climate extremes can impact the tourism and hospitality industry in urban areas. Events like hurricanes, heat waves, and wildfires can deter tourists from visiting urban destinations, leading to revenue losses for businesses and local economies. For example, the 2017 wildfires in California, USA, led to evacuations and closures of tourist attractions in cities like Los Angeles and San Francisco, resulting in lost revenues for hotels, restaurants, and tour operators. Besides, climate extremes, including heatwaves, air pollution episodes, and infectious disease outbreaks, can increase healthcare costs in urban areas. For example, the 2003 heatwave in Europe resulted in thousands of additional deaths and increased hospital admissions for heat-related illnesses, leading to higher healthcare costs for affected communities [136].

## 6. Mitigation and adaptation strategies

**Figure 8.**
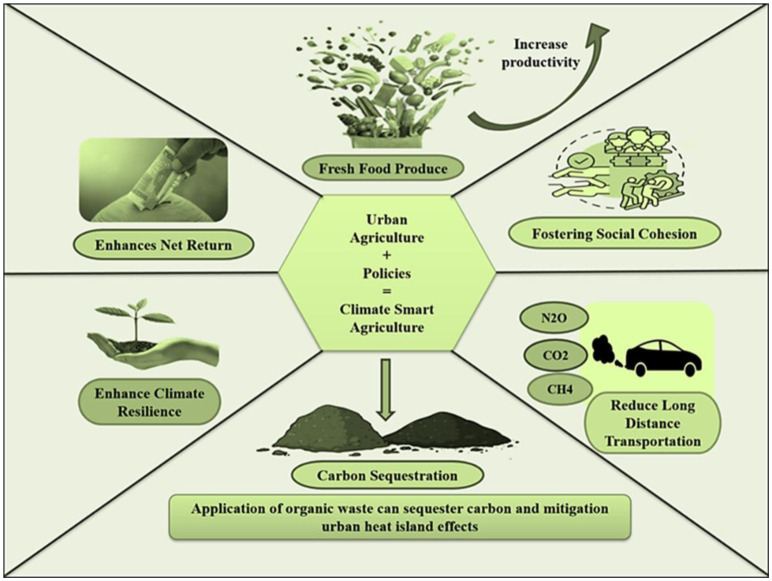
A climate-smart and sustainable ecosystem nurturing community and the environment.

Mitigation and adaptation strategies for the urban climate crisis are crucial for addressing the complex challenges of rapid urbanization and climate change [96]. A holistic approach that prioritizes both environmental sustainability and human well-being is essential. One key strategy is to promote sustainable urban design and planning, including green infrastructure such as parks, green roofs, and permeable surfaces that can help mitigate the urban heat island effect and improve air quality [46]–[48]. Ecological design and green infrastructure are essential to climate change adaptation by encouraging robust and sustainable solutions. Figure 8 conceptualizes climate-smart and sustainable ecosystem services through promoting green infrastructure.

The term “green infrastructure” describes networks of natural and semi-natural regions that are intentionally designed and maintained, as well as green areas found in urban settings [17]. Green roofs, green buildings, green walls, permeable pavements, bioswales, community parks, gardens, and vegetated surfaces are some of its components. Together, these components offer a number of advantages, such as the management of stormwater, the reduction of heat islands, the lowering of wind speeds, the reduction of energy consumption, the improvement of air quality, the preservation of biodiversity, the sequestration of carbon, and the maintenance of urban societies well-being. However, the exact scope of these benefits is up for debate [137]. Green infrastructure aids in the adaptation of cities and communities to climate change by assisting them in overcoming the obstacles presented by shifting climatic patterns. Green infrastructure improves water resource management and lowers the danger of floods by collecting and storing rainwater. By offering shade, evaporative cooling, and natural ventilation, it also aids in mitigating urban heat islands and lowers the amount of energy needed for cooling. Portland, Oregon, a city in the northwest of the United States, is one example of how using a green infrastructure design strategy to manage flood hazards may benefit the local community in many ways, both at the site and neighborhood level. The proactive steps made by the city's municipal administration in response to severe pressure on Portland's drainage system resulted in around 50 combined wastewater overflows into the Willamette River in 1990 [138]. They reduced the strain on the sewage system and reduced the negative effects on urban waterways by implementing a wide range of green infrastructure projects. These strategies include paying people to disconnect downspouts, rerouting stormwater to gardens, lawns, and ground infiltration, building green roofs to increase biodiversity in the area, and creating a network of recreational green spaces that also reduce into the Willamette. Implementing these initiatives results in a rise in the biodiversity of the surrounding area and an improvement in the streetscape's aesthetic appeal. Furthermore, an extensive and community-based drainage initiative in Vancouver, Canada, known as The Green Streets initiative, which funds roadside area improvement projects and encourages citizens to grow in their cities, has received strong support from the local government [139]. The main goal of ecological design is to include ecological concepts and procedures in the layout and creation of built environments. Ecological design aims to develop regenerative, sustainable systems that are in balance with the natural world. To promote resilience and flexibility, ecological design techniques consider a project's ecological, social, and economic components. Ecological design places a strong emphasis on tactics including conserving natural resources, utilizing native plant species, improving and restoring natural ecosystems, and encouraging biodiversity in the face of climate change. In India, cities like Chennai and Kochi are making the first steps toward becoming urban climate transition cities. By utilizing a wide range of initiatives, these Indian cities actively engaged in normative adaption integration. To strengthen urban resilience in the face of climate change concerns, they gave top priority to the repair and improvement of Blue-green infrastructures. Significantly, international cooperation organizations were crucial in helping the municipalities in these case studies, especially with the redevelopment of the Mullassery Canal in Kochi and the Buckingham Canal in Chennai, which represented a major turning point in creating a new regulatory framework [140]. Incorporating green infrastructures (green buildings, green roofs, and floors, green forests) into urban development can also improve habitat quality, increase landscape connectivity, and, in some situations, make it easier for vulnerable species to establish controlled populations [17]. These tactics boost ecosystem resilience while also offering a host of secondary advantages, such as better air and water quality, better habitats for animals, and greater community well-being. Cities may, therefore, better prepare for the future while boosting ecological health and human welfare through the incorporation of nature-based solutions into urban planning and design.

Additionally, implementing energy-efficient buildings/green buildings or retrofitting existing buildings by upgrading insulation, windows, and HVAC systems to improve energy efficiency and transportation systems by promoting the use of electric vehicles (EV) through incentives and the development of EV charging infrastructure can all reduce greenhouse gas emissions and enhance resilience to climate impacts [141]. Equitable interventions are also crucial, focusing on vulnerable communities disproportionately affected by climate risks. This involves enhancing social resilience through community-based adaptation initiatives and ensuring access to essential services such as clean water, healthcare, and adequate housing [142]. Furthermore, fostering inclusive governance processes that engage diverse stakeholders, including marginalized groups, in decision-making can lead to more effective and equitable climate action. Besides, investing in climate-resilient infrastructure, such as flood protection measures and disaster risk reduction strategies, is another critical aspect of adaptation [143]. This includes integrating nature-based solutions like wetlands restoration and coastal protection into urban planning to enhance ecosystem services and reduce the impacts of extreme weather events. Education and awareness campaigns can also play a vital role in fostering a culture of sustainability and resilience within urban communities, empowering individuals and organizations to take proactive measures to mitigate climate change and adapt to its impacts. Overall, a comprehensive approach that combines technical solutions with social equity and community engagement is essential for effectively addressing the urban climate crisis [144].

### 6.1. Policy interventions to address urban climate risks

Policy interventions are essential for addressing urban climate risks and fostering climate resilience in cities. These interventions encompass a range of measures such as climate action plans, land use planning, building codes and standards, transportation policies, green infrastructure mandates, water management regulations, climate resilience financing, community engagement, education, and adaptive governance structures. Climate action plans set clear targets and strategies for reducing greenhouse gas emissions and enhancing climate resilience, while land use planning ensures sustainable urban design and minimizes exposure to climate hazards [145]. Building codes and standards promote energy efficiency and climate-responsive building design, while transportation policies prioritize eco-friendly forms of transportation to lower emissions and enhance air quality [146]. Green infrastructure mandates integrate nature-based solutions into urban development, enhancing resilience and ecosystem services [17],[137]. Water management regulations focus on conservation, stormwater management, and flood prevention. Climate resilience financing supports climate projects and innovation, while community engagement and education build local resilience capacities. Adaptive governance structures facilitate coordination and collaboration among stakeholders. Together, these policy interventions play a crucial role in creating climate-resilient cities that can thrive in the face of climate change.

**Figure 9.**
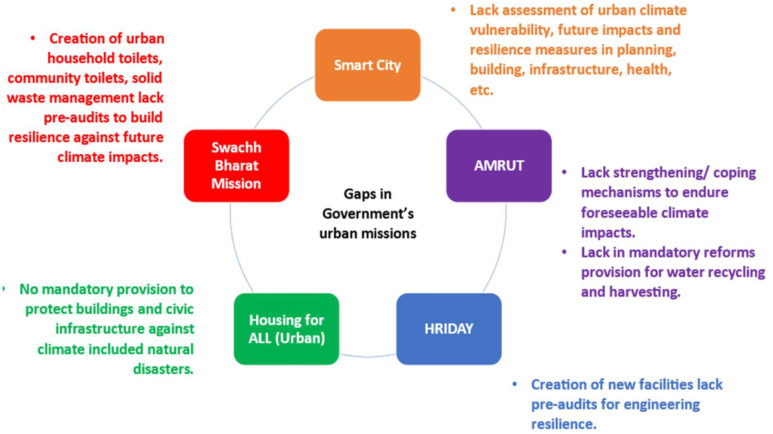
Strategic gaps in urban climate resilience components of the govt. of India's urban mission (*Adapted from* Sethi et al. [150])

For the past 100 years, integrating ecosystem services into urban planning has been an essential strategy in policy and planning for sustainable urban ecosystem services. Many crucial factors are involved in the planning and policy for sustainable urban ecosystem services. Primarily, it necessitates appreciating and acknowledging ecosystems' contributions to urban areas. This entails figuring out how much the numerous ecosystem services that natural systems offer are worth on an economic and social level. Policymakers and planners may give the preservation and restoration of ecosystems inside urban areas top priority by incorporating this knowledge into their decision-making processes. Second, sustainable urban ecosystem services planning places a strong emphasis on incorporating green infrastructure into urban development [45]. Furthermore, cooperation between stakeholders, such as governmental bodies, nonprofits, and commercial enterprises, is required for policy and planning for sustainable urban ecosystem services. Decision-making procedures are guaranteed to take into account and include a variety of viewpoints thanks to this collaborative method. Under the Kyoto Protocol, developed countries evolved climate change plans and approaches to expand the reach and coverage of the policies to include all sectors and all gases, as well as an overview of the policy instruments to combat climate change and fulfill their quantitative commitments [147]. A method for quantitatively climate change evaluation of mitigation policy instruments using integrated multicriteria analysis is comprised of three main components. First, a series of standards supported by further standards that describe the complex framework that decision-makers employ to choose and apply these tools. The second method is the analytical hierarchy process (AHP), which takes the preferences of three stakeholder groups into account when determining the weight coefficients for criteria and sub-criteria. Ultimately, each instrument evaluated for its performance under each distinct sub-criterion is given a grade using a multi-attribute theory (MAUT)/simple multiattribute ranking technique (SMART) procedure. The viability of the proposed strategy was investigated by assessing the overall results of the EU carbon-trading program in Denmark, Germany, Greece, Italy, Netherlands, Portugal, Sweden, and the United Kingdom [148]. Further, the development of the Energy and Climate Policy and Scenario Evaluation (ECLIPSE) model, a flexible integrated assessment tool for policy and scenario assessment related to energy and climate change [149]. In India, while several government initiatives have been taken in the sector of urban infrastructure like water supply, sanitation and waste management, urban mobility, public transport, health and education, electricity and affordable housing, robust IT and digitization, etc. [150], are not fully operational due to certain strategic gaps (Figure 9). Hence, it is important to address these gaps to make these government initiatives more robust and accessible to the public.

## 7. Future directions and research need

The intersection of urbanization and climate change poses significant challenges for both the environment and human well-being. Rapid urbanization, coupled with the effects of climate change, exacerbates environmental degradation, alters ecosystems, and threatens the livelihoods of millions of people worldwide. Understanding this complex nexus and devising effective strategies for mitigation and adaptation are paramount for sustainable urban development. In this section, we delve into the future directions and challenges of unraveling the urban climate crisis, focusing on emerging trends in urban climate research, technological innovations for climate mitigation and adaptation, and governance and policy frameworks for sustainable urban development.

### 7.1. Emerging trends in urban climate research

Urban climate research is evolving rapidly to address the multifaceted challenges of the urban climate crisis. One emerging trend is the integration of interdisciplinary approaches, combining insights from climatology, urban planning, ecology, sociology, and other fields. This holistic approach allows researchers to examine the interconnectedness of urbanization, climate change, and their impacts on the environment and human societies. For instance, a recent study highlighted the role of UHIs in exacerbating the urban climate crisis. UHIs, characterized by higher temperatures in urban areas compared to their rural surroundings, result from the absorption and retention of heat by built infrastructure. Researchers are employing advanced modeling techniques and remote sensing technologies to understand the drivers of UHIs and develop effective mitigation strategies, such as green infrastructure and cool roof initiatives [151]. Furthermore, there is growing interest in exploring the social dimensions of urban climate change. This includes understanding how climate-related hazards disproportionately affect vulnerable populations, such as low-income communities and marginalized groups. Integrating social equity considerations into urban climate research ensures that adaptation and mitigation strategies are inclusive and equitable.

### 7.2. Technological innovations for climate mitigation and adaptation

Advancements in technology play a crucial role in addressing the urban climate crisis by enabling innovative solutions for climate mitigation and adaptation. One promising innovation is the development of smart city technologies, which leverage data analytics, sensor networks, and artificial intelligence to optimize resource management, reduce energy consumption, and enhance urban resilience [152]. For example, the Internet of Things (IoT) is being utilized to monitor and manage urban infrastructure in real time, allowing cities to respond swiftly to environmental threats such as flooding and extreme heat events. Similarly, renewable energy technologies, such as solar panels and wind turbines, are becoming increasingly cost-effective and scalable, offering cities viable alternatives to fossil fuels [153]. Furthermore, nature-based solutions are gaining traction as effective means of climate adaptation in urban areas. Green infrastructure, including parks, green roofs, and urban forests, not only helps mitigate the urban heat island effect but also provides numerous co-benefits such as improved air quality, biodiversity conservation, and recreational opportunities [154].

### 7.3. Governance and policy frameworks for sustainable urban development

Effective governance and policy frameworks are essential for driving sustainable urban development and addressing the urban climate crisis. This entails collaboration between government agencies, civil society organizations, the private sector, and local communities to develop and implement inclusive and equitable policies. One promising approach is the concept of climate governance networks, which bring together diverse stakeholders to co-create and implement climate action plans at the local level. These networks facilitate knowledge sharing, capacity building, and collective decision-making, fostering a sense of ownership and accountability among stakeholders [155]. Moreover, there is growing recognition of the need for transformative policies that address the root causes of urban vulnerability to climate change. This includes measures to promote sustainable land-use planning, reduce greenhouse gas emissions, enhance resilience to extreme weather events, and ensure equitable access to resources and services [156].

**Figure 10.**
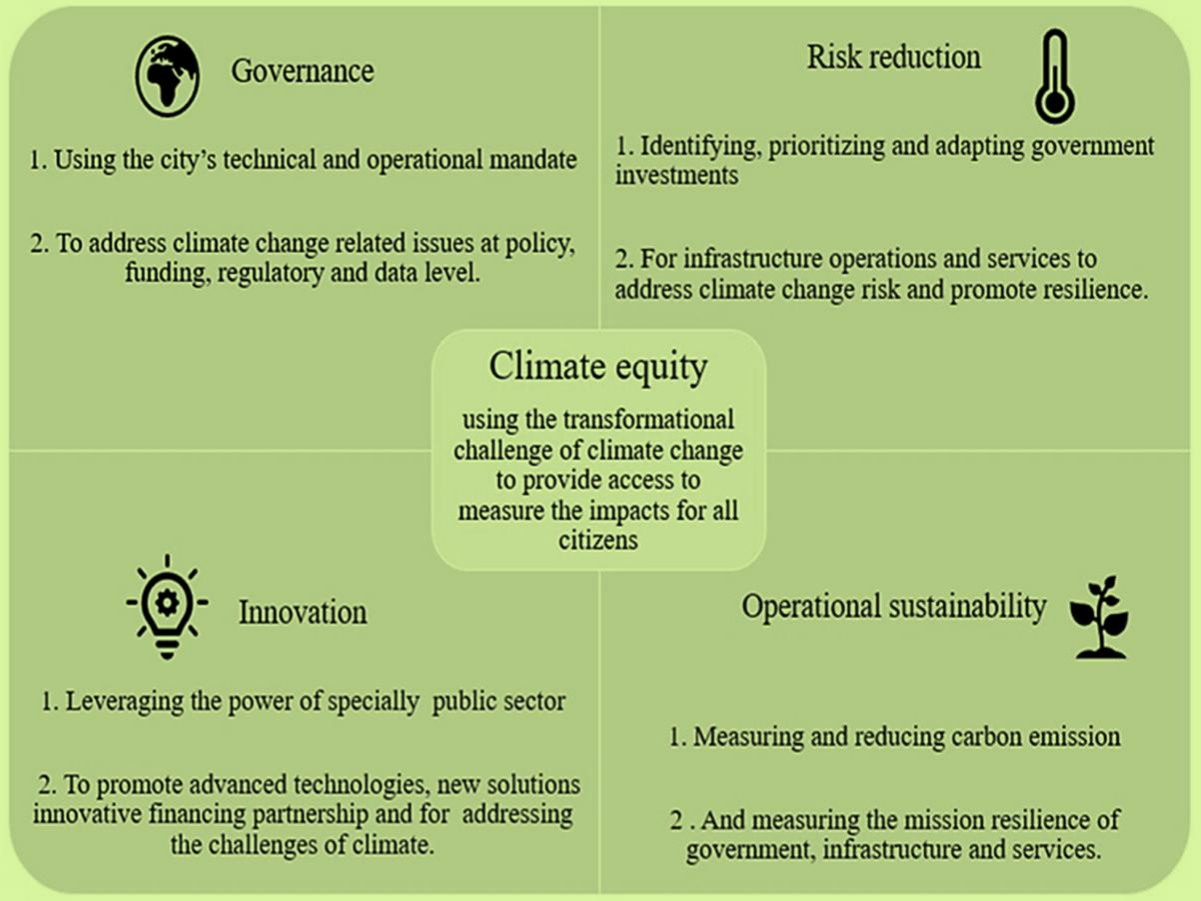
Five potential approaches to building climate-resilient cities.

Overall, the urban climate crisis poses profound challenges to the environment, human well-being, and sustainable development. Addressing these challenges requires concerted efforts from researchers, policymakers, technologists, and communities worldwide. By embracing interdisciplinary approaches, harnessing technological innovations, and fostering inclusive governance and policy frameworks, we can navigate the complexities of the urban climate crisis and build resilient, sustainable, and equitable cities for future generations (Figure 10).

## 8. Conclusions

We have examined the multifaceted impacts of urbanization on climate systems, highlighting the exacerbation of climate change through increased greenhouse gas emissions, heat island effects, and the alteration of local weather patterns. Conversely, climate change further complicates urban challenges by intensifying weather extremes, sea-level rise, and adverse health outcomes for urban populations. Global perspectives reveal that while the manifestations of urban climate crises vary across regions, common themes emerge. Rapid urbanization in developing countries often lacks the infrastructural resilience necessary to withstand climate impacts, leading to disproportionate burdens on vulnerable populations. In contrast, developed nations face the challenge of retrofitting existing urban infrastructure to mitigate and adapt to climate change, a task compounded by socio-political and economic considerations. Our key findings are summarized below, which are multifaceted and underscore the urgency of addressing the urban climate crisis through holistic and integrative approaches.

- ***Urbanization as a catalyst for climate change***: Rapid urbanization significantly contributes to climate change by increasing greenhouse gas emissions, particularly from transportation, industry, and energy sectors. Urban areas, which occupy less than 3% of the Earth's land, are responsible for over 70% of global CO_2_ emissions.
- ***Heat island effect***: UHIs are prevalent in densely populated cities, leading to higher temperatures than surrounding rural areas. This phenomenon exacerbates the effects of heatwaves, contributing to increased energy consumption, health issues, and mortality rates.
- ***Impact on air quality and public health***: Poor air quality in urban areas, driven by vehicle emissions, industries, and construction activities, poses severe health risks. The prevalence of respiratory and cardiovascular diseases is notably higher in urban populations, directly linking urbanization to public health crises.
- ***Water resources and urban flooding***: Urbanization impacts water resources by altering hydrological cycles, increasing surface runoff, and reducing groundwater recharge. The impervious surfaces in cities contribute to frequent and severe urban flooding, affecting infrastructure and livelihoods.
- ***Biodiversity loss and ecosystem degradation***: The expansion of urban areas leads to habitat destruction and fragmentation, resulting in significant biodiversity loss. Urban sprawl often encroaches on natural landscapes, reducing the ecosystem services essential for human well-being and environmental stability.
- ***Socioeconomic disparities***: Climate change and urbanization disproportionately affect marginalized and vulnerable populations. Socioeconomic disparities are accentuated, with poorer communities facing greater exposure to environmental hazards and having less capacity to adapt.
- ***Mitigation and adaptation strategies***: Effective mitigation and adaptation strategies are crucial in addressing the urban climate crisis. These include promoting green infrastructure, enhancing urban planning and design, increasing energy efficiency, and fostering community-based adaptation initiatives.
- ***Policy and governance***: Strong governance and policy frameworks are essential to manage the urbanization-climate nexus. Integrated policies that align urban development with climate resilience and sustainability goals are imperative for mitigating adverse impacts and promoting equitable growth.

Overall, the interplay between urbanization and climate change presents significant challenges and opportunities for innovation and resilience-building. Addressing the urban climate crisis requires a concerted effort from policymakers, urban planners, scientists, and communities. Emphasizing sustainable urban development, enhancing adaptive capacities, and fostering inclusive governance are critical steps towards mitigating the impacts of climate change on urban environments and improving the quality of life for urban populations worldwide. The review highlights the necessity of adopting a global perspective and collaborative approach to effectively unravel and address the complexities of the urban climate crisis.

## Use of AI tools declaration

The authors declare they have not used Artificial Intelligence (AI) tools in the creation of this article.

## References

[1] Grimm NB, Faeth SH, Golubiewski NE (2008). Global change and the ecology of cities. Science.

[2] Corburn J (2009). Cities, climate change and urban heat Island mitigation: Localising global environmental science. Urban Studies.

[3] Li Y, Smith AB, Johnson CD (2021). Understanding the urban heat island effect: A comprehensive study of temperature variations in urban areas. J Clim Stud.

[4] Rosenzweig C, Solecki W, Blake R, Rosenzweig C. (2020). Climate change and the urban future: Flooding and storm surges. Climate change and cities: Second assessment report of the urban climate change research network.

[5] Anguelovski I, Carmin J, Collier MJ (2021). Why green “climate gentrification” threatens poor and racially marginalized communities. P Natl Acad Sci.

[6] United Nations, Department of Economic and Social Affairs, Population Division (2019). World Urbanization Prospects: The 2018 Revision (ST/ESA/SER.A/420).

[7] IPCC (2018). Global warming of 1.5°C.

[8] World Bank (2021). Cities and climate change: An urgent agenda (English). Urban development series knowledge papers, no. 10.

[9] Global Commission on the Economy and Climate (2018). Unlocking the inclusive growth story of the 21st Century: Accelerating climate action in urgent times.

[10] Seto KC, Davis SJ, Christensen P, Edenhofer O., Pichs-Madruga R., Sokona Y. (2014). Human settlements, infrastructure, and spatial planning. Climate change 2014: Mitigation of climate change. Contribution of working group III to the fifth assessment report of the intergovernmental panel on climate change.

[11] Grimm NB, Chapin FS, Bierwagen B (2016). The impacts of climate change on ecosystem structure and function. Front Ecol Environ.

[12] Rosenzweig C, Solecki W, Romero-Lankao P (2018). Urban resilience to extreme weather events: Theoretical perspectives and practical insights. Environ Sci Policy Sustain Dev.

[13] McDonald RI, Kareiva P, Forman RTT (2019). Research gaps in knowledge of the impact of urban growth on biodiversity. Nat Sustain.

[14] Shi L, Zhang G, Chen Y (2016). Urban heat islands: Challenges towards sustainable heat mitigation. Climate.

[15] Díaz-Reviriego I, Fernandez-Giménez ME, Howard SE (2020). Climate change impacts on water-related illnesses in Sub-Saharan Africa. P Natl A Sci.

[16] World Bank (2023). World development indicators.

[17] Pandey B, Ghosh A (2023). Urban ecosystem services and climate change: A dynamic interplay. Front Sustain Cities.

[18] Zhang XQ (2016). The trends, promises and challenges of urbanisation in the world. Habitat Int.

[19] Elmqvist T, Setälä H, Handel SN (2015). Benefits of restoring ecosystem services in urban areas. Curr Opin Env Sust.

[20] Yang X, Shen J (2018). Urban land expansion and its driving factors in China: A comparative study of Beijing, Guangzhou, and Shanghai. Habitat Int.

[21] United Nations, Department of Economic and Social Affairs (2018). Population division “2018 revision of world urbanization prospects”.

[22] Gupta PK, Khan M (2020). Urbanization and urban growth in India: A critical review. J Reg Dev Planning.

[23] Couch C, Leontidou L, Arnstberg K (2008). Urban sprawl in Europe: Landscape, land-use change and policy.

[24] Meloche, Jean-Philippe, Wachter S. M., Zeuli K. A. (2015). Revitalizing American Cities. Revitalizing American Cities.

[25] Teixeira R, Cervero R (2019). Exploring the determinants of the suburbanization of jobs in the San Francisco Bay Area, 2005–2016. Urban Stud.

[26] Patel S (2017). Urban growth and sprawl in Delhi-NCR region: A comparative analysis. JURA.

[27] Roy A, Alsayed AA (2019). The political ecology of urban development: Infrastructure and everyday life in Dharavi, Mumbai. Int J Urban Reg Res.

[28] Liu X, Bae J (2018). Urbanization and industrialization impact of CO_2_ emissions in China. J Clean Prod.

[29] Leal Filho W, Perry P, Heim H (2022). An overview of the contribution of the textiles sector to climate change. Front Environ Sci.

[30] IEA (2022). Global energy review: CO_2_ emissions in 2021.

[31] Kumar JCR, Majid MA (2020). Renewable energy for sustainable development in India: Current status, future prospects, challenges, employment, and investment opportunities. Energy Sustain Soc.

[32] Wegrowski B (2019). Deforestation in the Amazon rainforest.

[33] Hinz R, Sulser TB, Huefner R (2020). Agricultural development and land use change in India: A scenario analysis of trade-offs between UN Sustainable Development Goals (SDGs). Earth's Future.

[34] Sovacool BK, Axsen J, Kempton W (2017). The future promise of vehicle-to-grid (V2G) integration: A sociotechnical review and research agenda. Annu Rev Environ Resour.

[35] Verma A, Harsha V, Subramanian G (2021). Evolution of urban transportation policies in India: A review and analysis. Transp in Dev Econ.

[36] Paravantis JA, Tasios PD, Dourmas V (2021). A regression analysis of the carbon footprint of megacities. Sustainability.

[37] Allen TR, Crawford T, Montz B (2019). Linking water infrastructure, public health, and sea level rise: Integrated assessment of flood resilience in coastal cities. Public Works Manag Policy.

[38] UN-Habitat (2020). World cities report 2020: The value of sustainable urbanization, The world cities report 2020, 418, UN-Habitat.

[39] Lankao PR, Nychka D, Tribbia JL (2008). Development and greenhouse gas emissions deviate from the ‘modernization’ theory and ‘convergence’ hypothesis. Clim Res.

[40] Anand P, Seetharam K (2010). Climate change and living cities: Global problems with local solutions. In Climate change and sustainable urban development in Africa and Asia.

[41] EEA (European Environment Agency) (2012). Climate change, impacts and vulnerability in Europe 2012. EEA Report No 12/2012, European Environment Agency, Copenhagen, Denmark.

[42] Hebbert M, Jankovic V, Webb B (2011). City weathers: Meteorology and urban design 1950–2020. University of Manchester, Manchester Architecture Research Centre.

[43] Carter JG, Connelly A, Handley J (2012). European cities in a changing climate: exploring climate change hazards, impacts and vulnerabilities. University of Manchester, Centre of Urban and Regional Ecology.

[44] Haase D, Frantzeskaki N, Elmqvist T (2014). Ecosystem services in urban landscapes: Practical applications and governance implications. Ambio.

[45] McPhearson T, Hamstead ZA, Kremer P (2014). Urban ecosystem services for resilience planning and management in New York City. Ambio.

[46] Nowak DJ, Greenfield EJ, Hoehn RE (2013). Carbon storage and sequestration by trees in urban and community areas of the United States. Environ Pollut.

[47] Agbelade AD, Onyekwelu JC (2020). Tree species diversity, volume yield, biomass and carbon sequestration in urban forests in two Nigerian cities. Urban Ecosyst.

[48] Klein LJ, Zhou W, Albrecht CM (2021). Quantification of carbon sequestration in urban forests. arXiv preprint.

[49] Pandey AK, Ghosh A, Rai K (2019). Abiotic stress in plants: A general outline. Approaches for enhancing abiotic stress tolerance in plants.

[50] Zhao H, Li G, Guo D (2021). Response mechanisms to heat stress in bees. Apidol.

[51] Xu Y, Ramanathan V, Victor DG (2018). Global warming will happen faster than we think. Nat.

[52] Mora C, Frazier AG, Longman RJ (2013). The projected timing of climate departure from recent variability. Nat.

[53] Grimmond S (2007). Urbanization and global environmental change: Local effects of urban warming. Geogr J.

[54] Kumar A, Pandey DAC, Khan ML (2020). Urban risk and resilience to climate change and natural hazards: A perspective from million plus cities on the Indian subcontinent. Techniques for disaster risk management and mitigation.

[55] Cheela VRS, John M, Biswas W (2021). Combating urban heat island effect—A review of reflective pavements and tree shading strategies. Build.

[56] Imam AU, Banerjee UK (2016). Urbanisation and greening of Indian cities: Problems, practices, and policies. Ambio.

[57] Arnfield AJ (2003). Two decades of urban climate research: A review of turbulence, exchanges of energy and water, and the urban heat island. Int J Climatol.

[58] Santamouris M, Cartalis C, Synnefa A (2015). On the impact of urban heat island and global warming on the power demand and electricity consumption of buildings—A review. Energy Build.

[59] Grover A, Singh RB (2015). Analysis of Urban Heat Island (UHI) in relation to Normalized Difference Vegetation Index (NDVI): A comparative study of Delhi and Mumbai. Environ.

[60] López-Guerrero RE, Verichev K, Moncada-Morales G (2022). How do urban heat islands affect the thermo-energy performance of buildings?. J Clean Prod.

[61] Kumari P, Garg V, Kumar R (2021). Impact of urban heat island formation on energy consumption in Delhi. Urban Clim.

[62] Hsu A, Sheriff G, Chakraborty T (2021). Disproportionate exposure to urban heat island intensity across major US cities. Nat Commun.

[63] Mohammad P, Goswami A (2021). Quantifying diurnal and seasonal variation of surface urban heat island intensity and its associated determinants across different climatic zones over Indian cities. GIScience Remote Sens.

[64] Zhan C, Xie M, Lu H (2023). Impacts of urbanization on air quality and the related health risks in a city with complex terrain. Atmos Chem Phys.

[65] Heidari H, Arabi M, Warziniack T (2021). Effects of urban development patterns on municipal water shortage. Front Water.

[66] Veena K, Parammasivam KM, Venkatesh TN (2020). Urban heat island studies: Current status in India and a comparison with the international studies. J Earth Syst Sci.

[67] Voelkel J, Hellman D, Sakuma R (2018). Assessing vulnerability to urban heat: A study of disproportionate heat exposure and access to refuge by socio-demographic status in Portland, Oregon. Int J Environ Res Public Health.

[68] Asian Development Bank (2022). Beating the heat: Investing in pro-poor solutions for urban resilience, Asian Development Bank (ADB).

[69] Wang K, Jiang S, Wang J (2017). Comparing the diurnal and seasonal variabilities of atmospheric and surface urban heat islands based on the Beijing urban meteorological network. J Geophys Res: Atmos.

[70] Lacke MC, Mote TL, Shepherd JM (2009). Aerosols and associated precipitation patterns in Atlanta. Atmos Environ.

[71] Hoornweg D, Sugar L, Gomez CLT (2020). Cities and greenhouse gas emissions: Moving forward. Urbanisation.

[72] da Encarnação Paiva AC, Nascimento N, Rodriguez DA (2020). Urban expansion and its impact on water security: The case of the Paraíba do Sul River Basin, São Paulo, Brazil. Sci Total Environ.

[73] Griggs G, Reguero BG (2021). Coastal adaptation to climate change and sea-level rise. Water.

[74] Kantamaneni K, Panneer S, Krishnan A (2022). Appraisal of climate change and cyclone trends in Indian coastal states: A systematic approach towards climate action. Arab J Geosci.

[75] Singh G, Khole M, Rase D (2015). Extreme rainfall events and urban floods in the growing cities of India. MAUSAM.

[76] Weiskopf SR, Rubenstein MA, Crozier LG (2020). Climate change effects on biodiversity, ecosystems, ecosystem services, and natural resource management in the United States. Sci Total Environ.

[77] Plane E, Hill K, May C (2019). A rapid assessment method to identify potential groundwater flooding hotspots as sea levels rise in coastal cities. Water.

[78] Speak A, Escobedo FJ, Russo A (2020). Total urban tree carbon storage and waste management emissions estimated using a combination of LiDAR, field measurements and an end-of-life wood approach. J Cleaner Prod.

[79] Carlan I, Haase D, Große-Stoltenberg A (2020). Mapping heat and traffic stress of urban park vegetation based on satellite imagery-A comparison of Bucharest, Romania and Leipzig, Germany. Urban Ecosyst.

[80] Kabisch N, Kraemer R, Brenck ME (2021). A methodological framework for the assessment of regulating and recreational ecosystem services in urban parks under heat and drought conditions. Eco People.

[81] Haase D, Hellwig R (2022). Effects of heat and drought stress on the health status of six urban street tree species in Leipzig, Germany. Trees, Forests and People.

[82] Bett B, Kiunga P, Gachohi J (2017). Effects of climate change on the occurrence and distribution of livestock diseases. Prev Vet Med.

[83] Ayanleye S, Udele K, Nasir V (2022). Durability and protection of mass timber structures: A review. J Build Eng.

[84] Landry SM, Koeser AK, Kane B (2021). Urban forest response to Hurricane Irma: The role of landscape characteristics and sociodemographic context. Urban For Urban Greening.

[85] Sabiiti EN, Katongole CB, Katuromunda S (2014). Building urban resilience: Assessing urban and peri-urban agriculture in Kampala, Uganda.

[86] Pandey B, Ghosh A (2022). Toxicological implications of fine particulates: Sources, chemical composition, and possible underlying mechanism. Airborne Particulate Matter: Source, Chemistry and Health.

[87] Green H, Bailey J, Schwarz L (2019). Impact of heat on mortality and morbidity in low and middle income countries: A review of the epidemiological evidence and considerations for future research. Environ Res.

[88] Schwarz N, Manceu AM (2015). Analyzing the influence of urban forms on surface urban heat islands in Europe. J Urban Plan Dev.

[89] Lu H, Gaur A, Krayenhoff ES (2023). Thermal effects of cool roofs and urban vegetation during extreme heat events in three Canadian regions. Sustain Cities Soc.

[90] Tsoka S, Tsikaloudaki K, Theodosiou T (2020). Urban warming and cities' microclimates: Investigation methods and mitigation strategies—A review. Energies.

[91] Yang J, Wang ZH, Kaloush KE (2015). Environmental impacts of reflective materials: Is high albedo a ‘silver bullet’ for mitigating urban heat island?. Renew Sustain Energy Rev.

[92] Masson V, Lemonsu A, Hidalgo J (2020). Urban climates and climate change. Annu Rev Environ Resour.

[93] Faurie C, Varghese BM, Liu J (2022). Association between high temperature and heatwaves with heat-related illnesses: A systematic review and meta-analysis. Sci Total Environ.

[94] Berardi U, Jafarpur P (2020). Assessing the impact of climate change on building heating and cooling energy demand in Canada. Renew Sustain Energy Rev.

[95] Burillo D, Chester MV, Pincetl S (2019). Electricity infrastructure vulnerabilities due to long-term growth and extreme heat from climate change in Los Angeles County. Energy Policy.

[96] IPCC (2022). Climate change 2022: Impacts, adaptation and vulnerability.

[97] Day JW, Gunn JD, Burger JR (2021). Diminishing opportunities for sustainability of coastal cities in the anthropocene: A review. Front Environ Sci.

[98] O'Donnell EC, Thorne CR (2020). Drivers of future urban flood risk. Philos Trans R Soc A.

[99] Merz B, Blöschl G, Vorogushyn S (2021). Causes, impacts and patterns of disastrous river floods. Nat Rev Earth Environ.

[100] Nazarnia H, Nazarnia M, Sarmasti H (2020). A systematic review of civil and environmental infrastructures for coastal adaptation to sea level rise. Civ Eng J.

[101] Tully K, Gedan K, Epanchin-Niell R (2019). The invisible flood: The chemistry, ecology, and social implications of coastal saltwater intrusion. BioScience.

[102] White N (2020). GMSL from TOPEX/Poseidon, Jason-1 and Jason-2 satellite altimeter data. Commonwealth Scientific and Industrial Research Organisation (CSIRO).

[103] Nicholls R, Zanuttigh B, Vanderlinden JP (2015). Developing a holistic approach to assessing and managing coastal flood risk. Coastal risk management in a changing climate.

[104] Abd-Elhamid HF, Abd-Elaty I, Hussain MS (2020). Mitigation of seawater intrusion in coastal aquifers using coastal earth fill considering future sea level rise. Environ Sci Pollut Res.

[105] NOAA (National Oceanic and Atmospheric Administration) (2020). Sea Level Trend 1993–2020.

[106] Revi A, Satterthwaite D, Aragón-Durand F, Field C.B., Barros V.R., Dokken D.J. (2014). Urban areas. Climate change 2014: Impacts, adaptation, and vulnerability. Part A: Global and sectoral aspects. Contribution of working group II to the fifth assessment report of the intergovernmental panel on climate change.

[107] Brook RD, Rajagopalan S, Pope CA (2010). Particulate matter air pollution and cardiovascular disease: an update to the scientific statement from the American Heart Association. Circ.

[108] Li T, Horton RM, Kinney P (2013). Future projections of seasonal patterns in temperature-related deaths for Manhattan. Nat Clim Chang.

[109] Debnath KB, Jenkins D, Patidar S (2023). Climate change, extreme heat, and South Asian megacities: Impact of heat stress on inhabitants and their productivity. ASME J Engi Sust Build Cities.

[110] World Health Organization (2021). Air pollution.

[111] Kaur R, Pandey P (2021). Air pollution, climate change, and human health in Indian cities: A brief review. Front Sustain Cities.

[112] Wieser AA, Scherz M, Passer A (2021). Challenges of a healthy built environment: Air pollution in construction industry. Sustainability.

[113] Rizwan S, Nongkynrih B, Gupta SK (2013). “Air pollution in Delhi: Its magnitude and effects on health”. IJCM: IAPSM.

[114] Pradeep BS, Gururaj G, Varghese M (2016). National mental health survey of India, 2016- Rationale, design and methods. PLoS One.

[115] Ventriglio A, Torales J, Castaldelli-Maia JM (2021). Urbanization and emerging mental health issues. CNS Spectrums.

[116] Gupta N, Aithal BH (2022). Effects of rising urban temperatures on the wellbeing of the residents: A case study of Kolkata Metropolitan Region. Int Rev Spat Plan Sustain Dev.

[117] Kalkstein LS, Smoyer KE (1993). The impact of climate change on human health: Some international implications. Experientia.

[118] Kolstad EW, Johansson KA (2011). Uncertainties associated with quantifying climate change impacts on human health: A case study for diarrhea. Environ Health Perspect.

[119] Ajjur SB, Al-Ghamdi SG (2022). Exploring urban growth–climate change–flood risk nexus in fast growing cities. Sci Rep.

[120] D'Souza RM, Becker NG, Hall G (2004). Does ambient temperature affect foodborne disease?. Epidemiol.

[121] Naumova EN, Jagai JS, Matyas B (2007). Seasonality in six enterically transmitted diseases and ambient temperature. Epidemiol Infec.

[122] Onozuka D, Hashizume M, Hagihara A (2010). Effects of weather variability on infectious gastroenteritis. Epidemiol Infec.

[123] Hashizume M, Armstrong B, Hajat S (2007). Association between climate variability and hospital visits for non-cholera diarrhoea in Bangladesh: Effects and vulnerable groups. Int J Epidemiol.

[124] Clayton S, Manning C, Krygsman K (2017). Mental health and our changing climate: Impacts, implications, and guidance.

[125] Hanna EG, McIver L (2018). Climate change: A brief overview of the science and health impacts for Australia. Med J Aust.

[126] Murari KK, Ghosh S, Patwardhan A (2015). Intensification of future severe heat waves in India and their effect on heat stress and mortality. Reg Environ Change.

[127] Kovats RS, Hajat S (2008). Heat stress and public health: A critical review. Annual Review of Public Health.

[128] Caminade C, Kovats S, Rocklov J (2014). Impact of climate change on global malaria distribution. Proc Natl Acad Sci U S A.

[129] Confalonieri UE, Marinho DP, Rodriguez RE (2009). Public health vulnerability to climate change in Brazil. Clim Res.

[130] Harlan SL, Pellow DN, Roberts JT (2015). Climate justice and inequality. Climate change and society: Sociological perspectives.

[131] Narzetti DA, Marques RC (2021). Access to water and sanitation services in Brazilian vulnerable areas: The role of regulation and recent institutional reform. Water.

[132] Wiryomartono B (2020). Capitalist agenda behind the seawall development in Jakarta bay, Indonesia: The marginalization of urban poor. Traditions and transformations of habitation in Indonesia.

[133] Hallegatte S, Green C, Nicholls RJ (2013). Future flood losses in major coastal cities. Nat Clim Chang.

[134] Colten CE, Kates RW, Laska SB (2008). Three years after Katrina: Lessons for community resilience. Environ: Sci Policy Susta Dev.

[135] Eriksen SH, Nightingale AJ, Eakin H (2015). Reframing adaptation: The political nature of climate change adaptation. Global Environ Change.

[136] Luber G, McGeehin M (2008). Climate change and extreme heat events. Am J Prev Med.

[137] Pataki DE, Carreiro MM, Cherrier J (2011). Coupling biogeochemical cycles in urban environments: Ecosystem services, green solutions, and misconceptions. Fron Ecol Environ.

[138] Hoyer J, Dickhaut W, Kronawitter L (2011). Water sensitive urban design: Principles and inspiration for sustainable stormwater management in the city of the future.

[139] Lancaster T (2006). Planning and implementation of sustainable stormwater management systems in the city of Vancouver: The green roof example [Doctoral dissertation].

[140] Sanchez FG, Govindarajulu D (2023). Integrating blue-green infrastructure in urban planning for climate adaptation: Lessons from Chennai and Kochi, India. Land Use Policy.

[141] Schoenefeld JJ, Hildén M, Schulze K (2022). Policy innovation for sustainable development. Handbook on the governance of sustainable development.

[142] Bardosh KL, Ryan SJ, Ebi K (2017). Addressing vulnerability, building resilience: community-based adaptation to vector-borne diseases in the context of global change. Infect Dis Poverty.

[143] Meng M, Dabrowski M, Stead D (2020). Enhancing flood resilience and climate adaptation: The state of the art and new directions for spatial planning. Sustainability.

[144] Hemani S, Das AK (2016). Humanising urban development in India: Call for a more comprehensive approach to social sustainability in the urban policy and design context. Int J Urban Sustain Dev.

[145] Waisman H, Bataille C, Winkler H (2019). A pathway design framework for national low greenhouse gas emission development strategies. Nat Clim Change.

[146] Lamsal P, Bajracharya S, Rijal H (2021). Guidelines for climate responsive building design in three regions of Nepal. Build Environ.

[147] Simeonova K, Diaz BH (2005). Integrated climate-change strategies of industrialized countries. Energy.

[148] Konidari P, Mavrakis D (2007). A multi-criteria evaluation method for climate change mitigation policy instruments. Energy Policy.

[149] Turton H (2008). ECLIPSE: An integrated energy-economy model for climate policy and scenario analysis. Energy.

[150] Sethi M, Sharma R, Mohapatra S (2021). How to tackle complexity in urban climate resilience? Negotiating climate science, adaptation and multi-level governance in India. PLoS One.

[151] Gartland LM, Luber G, Runkle J (2021). Urban heat islands: Disproportionate risks to underserved communities. Am J Public Health.

[152] Albino V, Berardi U, Dangelico RM (2015). Smart cities: Definitions, dimensions, performance, and initiatives. J Urban Technol.

[153] Ramaswami A, Russell AG, Culligan PJ (2016). Meta-principles for developing smart, sustainable, and healthy cities. Sci.

[154] Gómez-Baggethun E, Barton DN, Berry P (2013). The case of ecosystem services in urban resilience: A review of the literature. Cities.

[155] Bulkeley H, Castán Broto V (2013). Government by experiment? Global cities and the governing of climate change. T I Brit Geogr.

[156] Anguelovski I, Carmin J, Roberts D (2016). The urban climate justice agenda: A transformative action policy agenda. J Plan Educ Res.

